# Comprehensive comparison of Pacific Biosciences and Oxford Nanopore Technologies and their applications to transcriptome analysis

**DOI:** 10.12688/f1000research.10571.2

**Published:** 2017-06-19

**Authors:** Jason L Weirather, Mariateresa de Cesare, Yunhao Wang, Paolo Piazza, Vittorio Sebastiano, Xiu-Jie Wang, David Buck, Kin Fai Au

**Affiliations:** 1 Department of Internal Medicine, University of Iowa, Iowa City, IA, USA; 2 Oxford Genomics Centre, Wellcome Trust Centre for Human Genetics, University of Oxford, Oxford, UK; 3 Key laboratory of Genetics Network Biology, Collaborative Innovation Center of Genetics and Development, Institute of Genetics and Developmental Biology, Chinese Academy of Sciences, Beijing, China; 4 University of Chinese Academy of Sciences, Beijing, China; 5 Institute for Stem Cell Biology and Regenerative Medicine, Stanford University, Stanford, CA, USA; 6 Department of Obstetrics and Gynecology, Stanford University, Stanford, CA, USA; 7 Department of Biostatistics, University of Iowa, Iowa City, USA

**Keywords:** Third Generation Sequencing, PacBio, Oxford Nanopore Technologies, Transcriptome

## Abstract

*Background: *Given the demonstrated utility of Third Generation Sequencing [Pacific Biosciences (PacBio) and Oxford Nanopore Technologies (ONT)] long reads in many studies, a comprehensive analysis and comparison of their data quality and applications is in high demand. *Methods: *Based on the transcriptome sequencing data from human embryonic stem cells, we analyzed multiple data features of PacBio and ONT, including error pattern, length, mappability and technical improvements over previous platforms. We also evaluated their application to transcriptome analyses, such as isoform identification and quantification and characterization of transcriptome complexity, by comparing the performance of size-selected PacBio, non-size-selected ONT and their corresponding Hybrid-Seq strategies (PacBio+Illumina and ONT+Illumina). *Results: *PacBio shows overall better data quality, while ONT provides a higher yield. As with data quality, PacBio performs marginally better than ONT in most aspects for both long reads only and Hybrid-Seq strategies in transcriptome analysis. In addition, Hybrid-Seq shows superior performance over long reads only in most transcriptome analyses. *Conclusions: *Both PacBio and ONT sequencing are suitable for full-length single-molecule transcriptome analysis. As this first use of ONT reads in a Hybrid-Seq analysis has shown, both PacBio and ONT can benefit from a combined Illumina strategy. The tools and analytical methods developed here provide a resource for future applications and evaluations of these rapidly-changing technologies.

## Introduction

Third Generation Sequencing (TGS) emerged more than 5 years ago when Pacific Biosciences (PacBio) commercialized Single Molecule Real Time (SMRT) sequencing technologies in 2011 ^1^. Although TGS platforms have significant technical differences, they all generate very long reads (1–100kb) ^2– 5^, which is distinct from Second Generation Sequencing (SGS). Considering the paired-end information, the main SGS platform Illumina provides 50–600bp information from each DNA fragment; no SGS platforms provide >1000bp, including 454 sequencing, which generates the longest SGS reads ( ^~^700bp) ^6, 7^. Therefore, the short sequencing length limits the applications of SGS to large or complex genomic events, such as gene isoform reconstruction. TGS overcomes these challenging problems via long read lengths.

The most widely used TGS platforms [PacBio and Oxford Nanopore Technologies (ONT)] developed new biochemistry/biophysics methods to directly capture the very long nucleotide sequences from single DNA molecules. Other emerging TGS platforms (Moleculo ^8^ and 10X Genomics ^9^) are based on the assembly of short reads from the same DNA molecules to generate synthetic long reads (SLR). Herein, we focus on data features of PacBio and ONT and their applications to transcriptome analysis.

PacBio adopts a similar sequencing-by-synthesis strategy as Illumina sequencing, except PacBio captures a single DNA molecule and Illumina detects augmented signals from a clonal *population* of amplified DNA fragments. The error rate of raw PacBio data is 13–15%, as the signal-to-noise ratio from single DNA molecules is not high ^3^. To increase accuracy, the PacBio platform uses a circular DNA template by ligating hairpin adaptors to both ends of target double-stranded DNA. As the polymerase repeatedly traverses and replicates the circular molecule, the DNA template is sequenced multiple times to generate a continuous long read (CLR). The CLR can be split into multiple reads ("subreads") by removing adapter sequences, and multiple subreads generate circular consensus sequence ("CCS") reads with higher accuracy. The average length of a CLR is >10kb and up to 60kb, which depends on the polymerase lifetime ^3^. Thus, the length and accuracy of CCS reads depends on the fragment sizes. PacBio sequencing has been utilized for genome (e.g., *de novo* assembly, detection of structural variants and haplotyping) ^10^ and transcriptome (e.g., gene isoform reconstruction and novel gene/isoform discovery) ^11– 13^ studies.

ONT is a nanopore-based single molecule sequencing technology, and the first prototype MinION was released in 2014 ^14^. As compared to other sequencing technologies utilizing nucleotide incorporation or hybridization, ONT directly sequences a native single-stranded DNA (ssDNA) molecule by measuring characteristic current changes as the bases are threaded through the nanopore by a molecular motor protein. ONT MinION uses a hairpin library structure similar to the PacBio circular DNA template: the DNA template and its complement are bound by a hairpin adaptor. Therefore, the DNA template passes through the nanopore, followed by a hairpin and finally the complement. The raw read can be split into two “1D” reads (“template” and “complement”) by removing the adaptor. The consensus sequence of two “1D” reads is a “2D” read with a higher accuracy ^2^. Due to similar data features with PacBio, many researchers have utilized or are testing ONT in applications where PacBio has been applied.

PacBio and ONT platforms share the advantage of long read lengths, yet they also have the same drawback: higher sequencing error rate and lower throughput compared to SGS ^3, 14– 16^. High sequencing error rates pose challenges for single-nucleotide-resolution analyses, such as accurate sequencing of transcripts, identification of splice sites and SNP calling. Low throughput is an obstacle for quantitative analysis, such as gene/isoform abundance estimation. Although PacBio CCS and ONT 2D consensus strategies can reduce error rates, the corresponding read lengths become shorter and throughput becomes lower. Therefore, hybrid sequencing (“Hybrid-Seq”), which integrates TGS and SGS data, has emerged as an approach to address the limitations associated with analysis of TGS data with assistance of SGS data. For example, error correction of PacBio or ONT long reads by SGS short reads improves the accuracy and mappability of long reads ^17– 19^. Hybrid-Seq can be applied to genome assembly and transcriptome characterization and improve the overall performance and resolution ^11– 13, 17^.

The long read length of PacBio and ONT is very informative for transcriptome research, especially for gene isoform identification. In addition to human transcriptomes ^20– 22^, the PacBio transcript sequencing protocol, Iso-Seq, has been widely used to characterize transcriptome complexity in non-model organisms and particular genes/gene families ^23– 31^. In contrast, ONT has no standard transcript sequencing protocol and only a few pilot studies are publically available. Using MinION, Bolisetty *et al*. discovered very high isoform diversity of four genes in *Drosophila*, which illustrates the utility of ONT in investigating complex transcriptional events ^32^. Oikonomopoulos *et al*. also demonstrated the stability of ONT sequencing in quantifying transcriptome by analyzing an artificial mixture of 92 transcripts with Spike-In RNA ^33^. Compared to these studies using PacBio or ONT alone, Hybrid-Seq can reduce the requirement of data size and improve the output, especially for transcriptome-wide studies. For example, a series of Hybrid-Seq methods (IDP, IDP-fusion, IDP-ASE) have been developed to improve the transcriptome studies to isoform levels (e.g., gene isoform reconstruction, fusion genes and allele phasing) with higher sensitivity and accuracy, and achieve a more accurate abundance estimation, which has been demonstrated in human embryonic stem cells (hESCs) and breast cancer ^11– 13^.

Herein, we generated PacBio and ONT data from cDNA of hESCs. Using our tool AlignQC ( http://www.healthcare.uiowa.edu/labs/au/AlignQC/), we performed a comprehensive analysis and comparison of PacBio and ONT data, including the raw data (subreads and 1D “template” reads) and their consensus (CCS and 2D reads). PacBio sequencing was performed on size-selected libraries, as size selection is the manufacturer recommendation. ONT libraries were not size selected, because size selection was not standard practice at the time of sequencing and was not performed for ONT. Since these technologies follow different library preparation protocols, it is important to consider these steps as potential sources of variability just as the sequencing platforms themselves can introduce variability. Comparisons analyzed included error rate and error pattern, read length, mappability and abnormal alignments, as well as technology improvements between the latest sequencing models (PacBio P6-C4 and ONT R9) and previous versions (C2 and R7). We also validated and compared the capability of PacBio and ONT alone to study a gold standard set of spike-in transcripts. Then, we applied long read only and the corresponding Hybrid-Seq approaches to human transcriptome analyses, including isoform identification, quantification and discovery of complex transcriptome events. In addition to a comprehensive evaluation of the characteristics of the two main TGS data platforms, this work serves as a guide for applications of PacBio and ONT and the corresponding Hybrid-Seq for transcriptome analysis.

## Methods

### Cell culture and RNA extraction

Human embryonic stem cells (H1 cell line; WiCell) were cultured as previously described ^11^. In brief, cells were cultured in mTeSR1 (Stem Cell Technologies) on Matrigel matrix (BD). Cells were harvested between passages 50 and 55. Cells were fixed in 4% PFA for 10 minutes at room temperature and either incubated in blocking solution (2% FBS in PBS) or permeabilized in 0.2% Triton X-100 followed by incubation in blocking solution, where undifferentiated cells were verified by immunofluorescence (OCT4, NANOG, SSEA4, TRA-1-60, and TRA-1-81) as previous described ^34^. Briefly, the primary antibodies used in the study were as follows: anti-OCT4 (mouse; Santa Cruz; sc-5279; 1:500), anti-h-Nanog (rabbit; Cosmo Bio; REC-RCAB0004P-F; 1:200), anti-SSEA-3 (rabbit; Millipore; MAB4303; 1:500), anti-TRA-1-60 (mouse; Millipore; MAB4360; 1:500), anti-CD31 (R&D Systems), and anti-desmin (Thermo Fisher Scientific). Primary antibodies were diluted 1:200 in blocking solution, unless otherwise stated, and incubated overnight at room temperature. Secondary antibodies (goat or donkey; Invitrogen; Alexa 488 and Alexa 594; 1:5000) were incubated for two hours at room temperature. Pluripotency was confirmed by teratoma assay where three germ layers formed *in vivo* ^35^.

Total RNA was extracted using RNeasy Plus Mini Kit (QIAGEN). Agilent RNA 6000 Pico Kit (Agilent) was used to assess the RNA quality, and Qubit RNA BR Assay Kit (ThermoFisher Scientific) was used to quantify the extracted RNA. SIRV (Spike-in RNA Variant) E0 mixture (Lexogen, Batch No. 216652830) was added to the extracted total RNA (about 2.83% SIRVs in the final mixture).

### Library preparation and sequencing

For Illumina sequencing, TruSeq Stranded mRNA HT Sample Prep Kit (Illumina) was used to prepare the sequencing library by substituting the TruSeq barcoded adapter with Illumina Adapters (Multiplexing Sample Preparation Oligonucleotide Kit) and the PCR Primer Cocktail with Multiplex PCR primer 1.0 (5′-AATGATACGGCGACCACCGAGATCTACACTCTTTCCCTACACGACGCTCTTCCGATCT-3′) and custom index primer (5′-CAAGCAGAAGACGGCATACGAGAT[index]CAGTGACTGGAGTTCAGACGTGTGCTCTTCCGATCT-3’) as described previously ^36^. Sequencing was performed by Illumina HiSeq4000 with 150bp paired-end reads.

For PacBio sequencing, full-length cDNA and SMRTbell templates were prepared at the Centre of Genomic Research, University of Liverpool, following the Iso-Seq sample preparation protocol (Pacific Biosciences). For size selection, the full-length cDNA was fractioned into four contiguous size ranges (0–1kb, 1–2kb, 2–3kb, >3kb) on a Sage ELF (Sage Science) before constructing SMRTbell templates. Sequencing was performed by PacBio RS II using C4/P6 chemistry. The SMRT cell counts were 1, 4, 4 and 3 for 0–1kb, 1–2kb, 2–3kb and >3kb libraries, respectively.

For ONT sequencing, full-length cDNA was generated by the Smart-seq2 protocol, as described by Picelli *et al*. ^37^ using modified sequences for the TSO (5’-TTTCTGTTGGTGCTGATATTGCTGCCATTACGGCCrGrG+G-3’) and the Oligo-dT _30_VN (5’-ACTTGCCTGTCGCTCTATCTTCT _30_VN-3’) to allow amplification of the cDNA second strand with primers provided by ONT. The quality and size distribution of the cDNA was tested by a TapeStation Genomic DNA system (Agilent). For each ONT flowcell, 1 μg of double-stranded cDNA was converted in a Nanopore-compatible sequencing library using the Genomic DNA Sequencing Kit SQK– NSK007 (ONT), according to the manufacturer’s protocol with minor modifications. In detail, the ds-cDNA was subjected to a combined end repairing and dA-tailing step using the NEBNext Ultra™ II End Repair/dA-Tailing Module (New England BioLabs) and incubated for 30 min at 20°C followed by 30 min at 65°C. The reactions were purified with 0.4x volume Agencourt AMPure XP beads (Beckman Coulter), according to manufacturer’s instructions. The end-prepped cDNA was subsequently ligated to ONT leader- and HP-adapter using Blunt/TA Ligase Master Mix (New England BioLabs) with a 10 min incubation at room temperature. The ligated cDNA was annealed to a biotinylated tether oligo (ONT) that targets the hairpin-adapter (HP-adapter) by incubation for an additional 10 min at room temperature. The fragments with a HP-adapter ligated were selectively pulled down using Dynabeads MyOne Streptavidin C1 (Life Technologies). After washing the DNA-bounded beads to remove unbounded DNA, the captured cDNA library was released from the streptavidin beads by incubating the beads re-suspended in ONT Elution Buffer for 10 min at 37°C. The beads were then pelleted using a magnetic rack and the supernatant containing the library was recovered. The full-length cDNA library was sequenced on a MinION Mk 1B using a 48h sequencing protocol on R7/R9 chemistry flowcells.

### AlignQC software

Long reads require special considerations when accessing their quality; they have variable error rates and they are often size selected. These attributes make careful study of the alignments of long reads necessary to understand the quality and coverage of transcriptome sequencing. 

*Implementation:* AlignQC ( http://www.healthcare.uiowa.edu/labs/au/AlignQC/) is designed to provide comprehensive quality assessment for TGS long read sequencing alignment data by three layers: (1) basic statistics of the data, including read length, alignment and coverage across all chromosomes; (2) error pattern analysis if a reference genome is provided; (3) transcript-related statistics if a gene annotation is provided. AlignQC takes the standard BAM format file as the input, outputs XHTML format file for easy visualization, and provides links to access all analysis results.

For basic statistics of the data, AlignQC parses the CIGAR string and SEQ fields from the BAM file. Multiple alignment paths can be reported for each read, but only the longest aligned path is used in error rate calculations and annotation analyses. For alignment statistics, if two or more alignment paths are reasonably spaced across the read, and can together generate a longer alignment, they will be combined and classified as: (a) a gapped alignment of a gene if paths occur within close proximity to each other on the same strand; (b) a trans-chimeric alignment if paths occur on different loci; (c) a self-chimeric alignment if paths align to an overlapping genomic position; otherwise, the read is defined as (d) a single alignment.

For the error pattern analysis, AlignQC compares the aligned reads to the provided reference genome. Based on the difference between aligned reads and reference genome, it estimates the error rates of total and different error types, including substitutions, insertions, and deletions. The overall error rates are calculated by sampling alignments until at least 1 million aligned bases have been included. Context-specific error pattern is analyzed by randomly sampling the best alignments until each individual context has been observed at least 10,000 times.

For transcript related statistics, AlignQC firstly annotates the aligned reads according to their overlap with provided genes/transcripts. A read is assigned to a reference transcript if it can cover the first and last exons with any length, and the internal exons with ≥ 80% length. When multiple exons are present and both the read and the reference transcript have the same consecutive exons, the match is called as a “full-length” match, otherwise, it is referred to as a “partial” match.

*Operation:* AlignQC usage can be divided in to report generation, and report viewing. Report generation requires a Linux operating system with coreutils (version 8.6 or newer) and python (2.7 or newer); both are present in most current Linux releases. R must be installed (tested with version 3.3.0; https://www.r-project.org/). At least 16GB of RAM is recommended to run AlignQC. A full analysis of an alignment from a PacBio SMRT cell containing 107,960 molecules was processed by 4 threads in 32m21.307s. A full analysis of an alignment from an ONT R9 flow cell containing 387,810 molecules required 52m22.163s.

Report viewing can be done through any modern web browser and does not require any specific operating system. The primary output of AlignQC is an XHTML format report. Analysis files are embedded in the report; these include high quality plots and the long read mappings that are compatible with the UCSC genome browser ^38^. These reports can serve as both an analysis archive and a convenient means to share results.

### Short read and long read data processing and alignment

For Illumina short reads, the quality was assessed by FastQC. The sequencing adapters were trimmed by 9 bases on the 5’ end and adapters were removed by cutadapt ^39^ with the parameter “-a AGATCGGAAGAG -A AGATCGGAAGAG -m 50”. Short read alignment was performed by HISAT with default parameters. For SIRV, the reference genome (SIRV_151124a.fasta; https://www.lexogen.com/wp-content/uploads/2015/11/SIRV_Sequences_151124.zip; Supplementary Table 1) was provided by Lexogen. For the hESCs analysis, the reference genome was downloaded from UCSC (hg38 assembly; GCA_000001305.2; http://hgdownload.cse.ucsc.edu/goldenPath/hg38/chromosomes/).

For PacBio, the subreads and CCS reads were extracted using SMRT Analysis software (version 2.3.0; http://www.pacb.com/products-and-services/analytical-software/smrt-analysis/). For technical comparisons, CCS and subreads are used as referred. For the transcriptome analyses, PacBio data sets were comprised of “best reads”. These were constructed with the goal of 1) having each molecule represented in the dataset once and only once and 2) choosing the best-quality read of each molecule for transcriptome analysis. Below is the priority order of reads to be selected as the "best read" for each molecule in different analysis strategies:

For “PacBio only” strategy, “best reads” were selected by using 1) the best aligned CCS reads (determined by the number of mapped bases in the read), or if no CCS read or alignment was available, 2) the best aligned subread. For the “PacBio+Illumina” Hybrid-Seq analysis the “best read” is 1) the best aligned CCS reads with >2 passes and an accuracy greater than 95 (estimated by SMRT Analysis software); otherwise 2) the best aligned CCS reads corrected by short reads; otherwise 3) the best aligned subread.

For ONT, the template, complement and 2D reads were extracted by poretools software (version 0.5.1; https://poretools.readthedocs.io/en/latest/). For technical comparisons 2D reads and 1D template strand reads are referred as 2D and 1D, respectively. For the transcriptome analyses, a “best reads” set analogous to PacBio was used. For the “ONT only” strategy, “best reads” were selected by 1) the best aligned 2D reads, otherwise 2) the best aligned 1D template strand reads, otherwise 3) the best aligned 1D complement strand reads. For “ONT+Illumina” Hybrid-Seq strategy, the “best read” was selected by the same order after error correction by short reads.

For PacBio and ONT long read alignment, GMAP ^40^ (version 2016-06-30) was used with the parameter “-n 10”.

### Isoform identification in SIRVs by Illumina, PacBio, ONT

The SIRV (Lexogen) transcriptome, which consists of 69 transcripts, mimics 7 human model genes and includes all kinds of complex alternative splicing events. SIRV is useful to assess the performance of sequencing technology applied to studying human transcriptome. This study used the SIRV E0 mix (Batch No. 216652830, in which isoform SIRV502 is missing) with 68 RNA variants. The concentration ratio is identical for each isoform. Meanwhile, Lexogen also provides three types of annotation libraries: “corrected”, with all 68 truly-expressed isoforms; “insufficient”, including 43 of 68 truly-expressed isoforms; and “over-annotated”, with 68 truly-expressed isoforms and an additional 32 falsely-expressed isoforms.

When illustrating the performance of Illumina short reads on isoform identification, reference-guided assembly software StringTie ^41^ (version 1.3.0) with default parameters was used, based on three different SIRV annotation libraries above. For all SIRV isoforms, we classified them into two groups: 1) true positive if the isoform was annotated by SIRV “correct” annotation library; and 2) false positive if not. The numbers of true positive and false positive assembled isoforms were counted when using three SIRV annotation libraries in StringTie, respectively.

When illustrating the performance of PacBio and ONT long reads on isoform identification, an isoform was considered identified when at least one long read was uniquely aligned to this isoform.

### Isoform identification in hESCs by PacBio, ONT and Hybrid-Seq

Gencode (version 24) gene annotation library ( https://www.gencodegenes.org/; Supplementary Table 1) was used for isoform detection. AlignQC was used to identify isoforms annotated by Gencode (version 24). Briefly, for isoforms with only one exon (singleton isoform), if 90% of the isoform length could be covered by at least one long read, it was considered identified. For isoforms with multiple exons (multi-exon isoform), we required at least one long read that covered the first and last exons and ≥ 80% mutual overlap of each internal exon.

Notably, for Hybrid-seq (PacBio+Illumina and ONT+Illumina) strategies, we combined the results mentioned above and the output of IDP ^11^ (version 0.1.9), which is a tool specifically for isoform detection and prediction by Hybrid-seq data. The primary parameters of IDP were “Njun_limit=10, Niso_limit=100, and FPR=0.05”, using Gencode (version 24) as the primary reference, and a comprehensive transcript reference from the combination of Gencode (version 24), RefSeq (UCSC version 2015-06-03; http://hgdownload.cse.ucsc.edu/goldenPath/hg38/database/refFlat.txt.gz; Supplementary Table 1) and ESTs (downloaded from UCSC genome browser; http://hgdownload.cse.ucsc.edu/goldenPath/hg38/database/all_est.txt.gz; Supplementary Table 1).

For novel isoform identification, the output of IDP with the same parameters was used.

When investigating the accuracy of splice sites/exon boundaries within the multi-exon isoforms, we calculated the relative distance between known splice sites annotated by Gencode and detected splice sites by four strategies.

For repetitive element analysis, the lower-case sequence marked by RepeatMasker and Tandem Repeats Finder tools was used from the reference genome (UCSC hg38; http://hgdownload.cse.ucsc.edu/goldenPath/hg38/chromosomes/; Supplementary Table 1). For each isoform, the proportion of repetitive element sequence was calculated.

### Isoform abundance estimation by PacBio, ONT and Hybrid-Seq

The isoforms identified by 7 strategies (1. use Illumina data by StringTie with the “correct” SIRV annotation library; 2. use Illumina data by StringTie with the “insufficient” SIRV annotation library; use Illumina data by StringTie with the “over-annotated” SIRV annotation library; 4. use PacBio data with the “correct” SIRV annotation library; 5. use ONT data with the “correct” SIRV annotation library; 6. use PacBio+Illumina data with the “correct” SIRV annotation library; and 7. use ONT+Illumina data with the “correct” SIRV annotation library) were used to perform isoform abundance estimation.

The relative expression percentage (REP) of each isoform was calculated. Expected REP is 1/68.

For three Illumina-only strategies (Illumina data with the “correct” SIRV annotation library, Illumina data with the “insufficient” SIRV annotation library and Illumina data with the “over-annotated” SIRV annotation library), the TPM (transcripts per million) value from RSEM with default parameter was used to calculate the REP.

For two long read only strategies (PacBio data with the “correct” SIRV annotation library and ONT data with the “correct” SIRV annotation library), the read count from AlignQC was used to calculated the REP.

For two Hybrid-Seq strategies (PacBio+Illumina data with the “correct” SIRV annotation library and ONT+Illumina data with the “correct” SIRV annotation library), only the Illumina short read data was used to run RSEM with default parameters. The TPM (transcripts per million) value from RSEM was used to calculate the REP.

To compare the estimation error of 7 strategies, the euclidean distance between expected REP and estimated REP was calculated.

### Complexity analysis of the hESC transcriptome

For alternative splicing analysis, LESSeq ( https://github.com/gersteinlab/LESSeq) was used, following its instructions. We required a minimum frequency of each alternative event >10%.

### Functional analysis of identified isoforms in hESCs

For the prediction of protein coding capability of novel isoforms, GeneMarkS-T (version 5.1; http://exon.gatech.edu/GeneMark/) with default parameters was used. For gene enrichment analysis, DAVID (version 6.8) ^42^ was used.

## Results

### Read length of PacBio and ONT data

The mappable length is a good representation of the useful length of long reads. The median mappable lengths of PacBio data are 1,299bp and 1,464bp for subreads and CCS reads, respectively. ONT data are slightly longer, with median lengths of 1,602bp and 1,754bp for 2D and 1D reads, respectively ( Table 1), although size selection was performed in PacBio, but not in ONT (Methods).

**Table 1.** Statistics of mappable length and error rates of PacBio and ONT long reads.

Read type	Mappable length (bp)				Error rate (Proportion of overall error) (%)			
	Mean	Median	Standard deviation	Maximum	Overall	Insertion	Deletion	Mismatch
PacBio CCS	1772	1464	1132	8006	1.72	0.087 (5.06)	0.34 (19.48)	1.30 ( **75.46**)
PacBio subread	1570	1299	1076	16040	14.20	5.92 ( **41.71**)	3.01 (21.17)	5.27 (37.12)
ONT 2D	1861	1754	882	9126	13.40	3.12 (23.30)	4.79 (35.70)	5.50 ( **40.99**)
ONT 1D	1695	1602	824	9345	20.19	2.93 (14.51)	7.52 (37.24)	9.74 ( **48.25**)

The fractions of each error types are in parenthesis. The fractions of the most predominant error types in each data are in bold.

**Figure 1.**
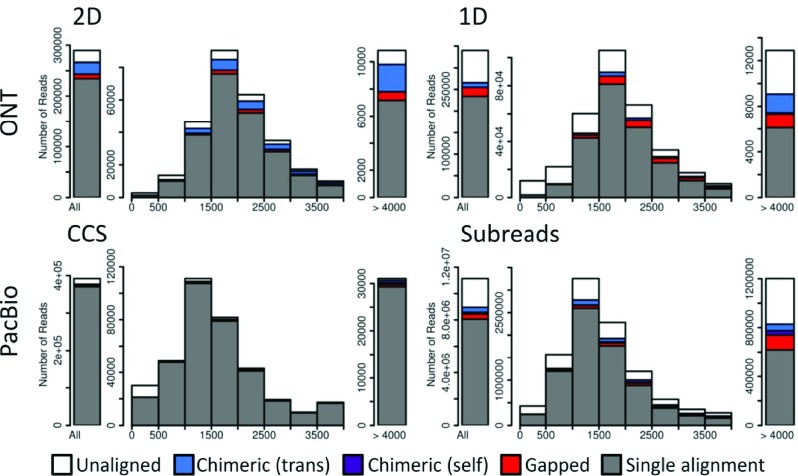
Length distribution of reads. The length distribution of Oxford Nanopore Technologies (ONT) 2D and 1D reads (top) and Pacific Biosciences (PacBio) CCS and subreads (bottom). Aligned reads are color-coded to indicate fraction of reads that are: single best alignments (gray), gapped alignments consisting of multiple paths (red), self-chimeric alignments (purple) where different read segments map to overlapping sequences, and trans-chimeric alignments (blue) where read segments map to different loci; white color represents unaligned reads. The leftmost bar represents all reads, the middle portion reads from 0–4kb in length, and the rightmost are reads greater than 4kb. PacBio libraries were size-selected, while ONT libraries were not; this provides PacBio with a larger proportion of longer reads. The total number of reads sequenced and the number of aligned reads from each sequencing platform are available in Supplementary Table 2.

The overall length distributions of the raw data and consensus data for both PacBio and ONT (subreads vs. CCS and 1D vs. 2D) are similar, while the differences between PacBio and ONT are more remarkable ( Figure 1). Compared to ONT, the length distribution of PacBio data skews to the left, with many reads <1kb, which may be caused by a short size-selected fraction (<1kb) of cDNA library (see Methods, Figure 1 and Supplementary Figure S1). In addition, CCS reads have a large proportion of very long reads (>3.5kb), as the high quality of CCS reads guarantee the alignment of the full length while the other reads (e.g., subreads) are partially aligned.

ONT R9 and the previous sequencing platform R7 have similar length distributions ( Figure 1, Supplementary Figure S2.1 and Supplementary Figure S2.2), while the yield of R9 is much higher (204,891±61,389 vs. 61,799±42,393 molecules were sequenced and mappable per R9 and R7 per flow cells, respectively). Thus, R9 provides a more stable and higher throughput, which will allow broader applications of ONT data ( Supplementary Table 2). The length distribution of the previous PacBio C2 sequencing data skews to a shorter length, compared to P6-C4. The yield of P6-C4 increased (76,597±23,387 vs. 21,827±9,707 molecules were sequenced and mappable per P6-C4 and C2 per SMRT cells, respectively). Overall, the yield per flow cell of ONT is much higher than PacBio, because each nanopore can sequence multiple molecules, while the wells of PacBio SMRT cells are not reusable. In addition, the PacBio read lengths in each SMRT cell are consistent with the sizes selected, so the size-selection protocol works well for PacBio data ( Supplementary Figure S1).

### Mappability of PacBio and ONT data

**Figure 2.**
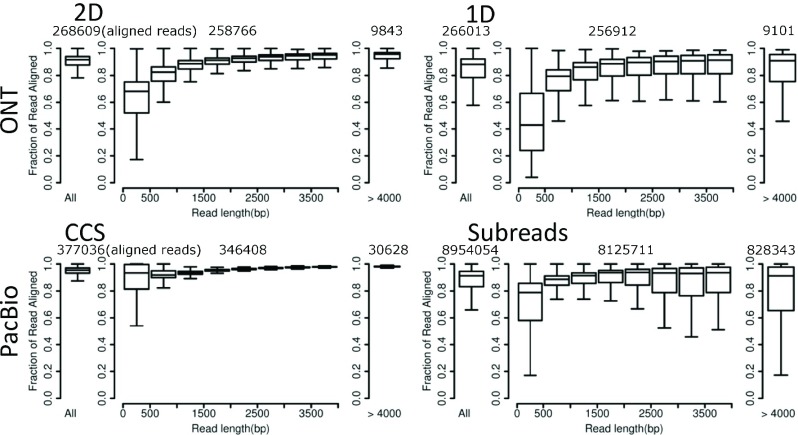
Mappability of different length bins. The leftmost bar represents the fraction of the mappable read length out of the total read length for all reads. The middle section shows the mappable fraction for 500bp increments ranging from 0–4kb read lengths, and the rightmost bar represents the mappable fraction of reads greater than 4kb. ONT: non-size-selected Oxford Nanopore Technologies reads; PacBio: size-selected Pacific Biosciences reads. The numbers of aligned reads contributing to the box plots in each panel are listed above each panel: total aligned reads, aligned reads <4kb and aligned reads >4kb (from left to right).

Mappability of long reads is necessary to confirm repetitive elements, gene isoforms and gene fusions ^11, 12, 21^. PacBio subreads and ONT 1D reads have similar rates of aligned reads (80.41% and 78.24%) and bases (81.80% and 81.03%) to the reference genomes ( Figure 2). However, a higher proportion of PacBio CCS reads (96.15%) and bases (95.07%) can be aligned than ONT 2D reads (92.05% and 87.37%), while both are higher than their corresponding raw data (subreads and 1D). Thus, generation of consensus sequences truly improves data quality. As 2D reads only sequence target molecules twice, it is expected to have lower quality than CCS with multiple subreads.

For all types of data, we consistently observe that short read lengths (<500bp) have low alignment rates. This is likely due to a larger portion of adapter and linker sequences in this short-length data bin. In addition, although a large fraction of ONT data are defined as “fail” reads during the data pre-process and filtered out, the alignment rates are as high as 65.74% and 50.95% for 2D fail reads and 1D fail reads, respectively. These findings indicate that parts of the fail reads are informative and should be rescued (e.g., by error correction) to increase throughput.

The mappability of PacBio data is similar between the C2 and P6-C4 chemistries, while the ONT 1D reads in R9 have almost doubled the proportion of aligned bases relative to R7 (81.03% vs. 44.43%). However, the alignment rate of R9 1D reads is surprisingly slightly worse than the previous R7 data (78.24% vs. 82.19%). The improvements in total bases aligned is likely attributable to improvements in raw data quality, while relaxing criteria for calling 1D reads in R9 may explain the slight drop in the overall alignment rate. The slight drop in the alignment rate accompanies a largely improved throughput of 1D reads per cell for R9 compared to R7 (181,599±54,331 vs. 55,366±26,371).

### Chimeric and gapped alignments of PacBio and ONT data

Long reads generated from gene fusions or trans-splices can be aligned to separated genomic loci, which are denoted as "trans-chimeric". Since hESCs contain very few fusion events or trans-splices, trans-chimeric reads are likely due to library preparation artifacts. 2D data contain 8.05% trans-chimeric reads, while 1D data contain surprisingly less (3.16%). Considering they are from the same data and library preparation, the lower trans-chimeric frequency in 1D reads may be due to the very low mappability of some error-prone regions. ONT data have particularly higher trans-chimeric rates in very long reads (>4kb) ( Figure 1). PacBio CCS reads have far less trans-chimeric alignments (0.93%), while 1D reads and subreads are of similar trans-chimeric fractions (3.47% vs 3.16%). Therefore, the library preparation artifact is not negligible in TGS data, and the trans-chimeric reads in non-tumor samples should be filtered before further usage. In addition, two fragments of a long read may be aligned to the same genomic locus, denoted as "self-chimeric", because of the failure of removing adaptor sequences from the raw data (e.g., PacBio CLR). Overall, self-chimeric proportion is much smaller than trans-chimeric. The chimeric reads may cause an overestimate of the lengths of DNA molecules.

Since some regions of long reads may be particularly error-prone, long reads may be aligned as separated fragments. With careful analysis, these "gapped alignments" can be used similarly with the paired-end Illumina reads. Corresponding to the high error rate, more ONT data are gapped alignments (1D: 6.10% and 2D: 2.98%) than PacBio (subreads: 3.45% and CCS: 0.48%). This rate is even more severe in the previous ONT R7 chemistry, especially for 1D reads (30.82%), while the difference between PacBio C2 and P6-C4 data is much smaller.

### Error pattern of PacBio and ONT data

Whereas mappability is a metric of the fraction of useful reads, error rate and error pattern measure the quality of the data, which have a strong effect on single-nucleotide resolution analysis (e.g., SNP calling and splice detection) and design of error correction algorithms. The error rate of PacBio CCS reads is as low as 1.72%. The 14.20% error rate of subreads is consistent with previous reports (Korlach J. Understanding Accuracy in SMRT Sequencing. Pacific Biosciences; http://www.pacb.com/wp-content/uploads/2015/09/Perspective_UnderstandingAccuracySMRTSequencing1.pdf.) and is similar with ONT 2D data (13.41%). However, 1D reads have a 20.19% error rate ( Table 1). Thus, the raw data and the consensus sequence of PacBio data are of higher base quality than corresponding ONT data.

Moreover, the composition of PacBio and ONT errors are different. Mismatches are the major errors in both ONT data (2D: 40.99% and 1D: 48.25%), and the proportion of deletions are also as high as >35% ( Table 1). Thus, insertions are the least common errors in ONT. Insertions are also the least common in PacBio CCS reads, whereas mismatches are more predominant (75.70%), though the absolute error rate is fairly low. Conversely, the rate of insertions in subreads is the highest (41.71%), and mismatches are at a similar level (37.12%). Thus, insertions and deletions together (“indels”) contribute to most errors with the exception of CCS reads.

**Figure 3.**
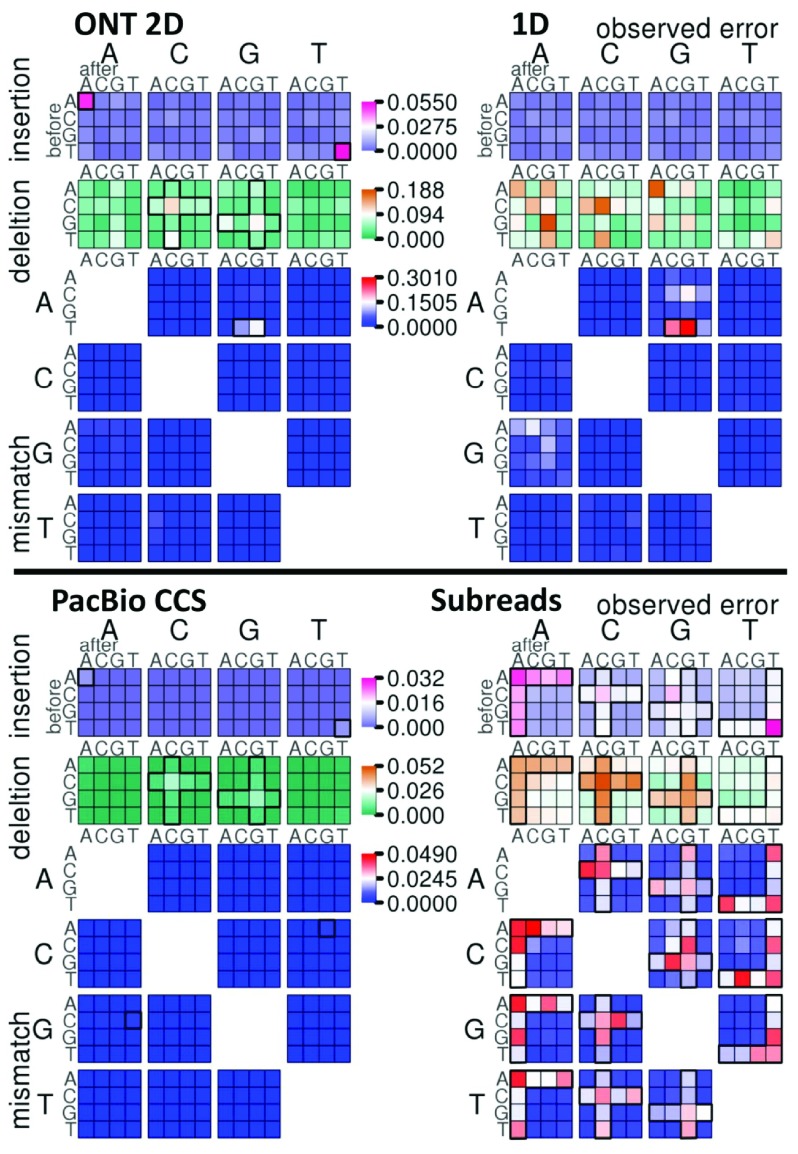
Context-specific errors. Context specific-errors are shown for Oxford Nanopore Technologies (ONT) 2D and 1D reads (top), and Pacific Biosciences (PacBio) CCS and subreads (bottom). The error types shown are insertions, deletions and mismatches. For insertions, the large base above the plot indicates the inserted base, and for deletions, the deleted base. For mismatch errors, the large base to the left indicates the expected reference base, and the large base above indicates the base observed in the read. A block of color tiles shows the error frequency within specific contexts for each error; the small base to the left of the tiles indicates the base preceding the error, and the small base above is the base following error. Error frequency is plotted on separate scales for insertions, deletions, and mismatches. Homopolymer error patterns are highlighted with a bold cross- or L-shaped outlines in the ONT 2D, PacBio CCS and PacBio Subreads plots. Context-specific insertions and mismatches of interest in the ONT 1D, 2D and PacBio CCS reads are highlighted by a bold outlines. For a better contrast of lower error rate in PacBio CCS reads and ONT 2D reads, Supplementary Figure S4 displays each result with its own scale.

PacBio base calling is based on distinguishing signals from the neighborhood background; ONT relies on the current signal change from the five upstream bases. Therefore, their errors may both have context-specific patterns. As the predominant error type in CCS reads, mismatches mostly arise from two context-specific events: C **G**->C **A** and **C**G-> **T**G ( Figure 3); however, these mismatches are likely alignment errors rather than sequencing errors as they are also observed in the alignments of high-quality Illumina data and simulation data ( Supplementary Figure S3). The mismatch T **A**G->T **G**G is most striking in both ONT 2D and 1D reads, followed by T **A**C->T **G**C, while the other mismatches are far less frequent ( Figure 3). In contrast, the mismatches in subreads show a clear “loose homopolymer pattern”: the base is more likely mis-called as either the upstream or downstream base (“cross shape” in Figure 3). The same homopolymer pattern also exists in the indels in subreads: 46.07% indels are in a homopolymer ( Figure 3). The indels prefer to occur in homopolymers in CCS and 2D reads as well, with 85.46% and 39.40% in homopolymers, respectively. In addition, both CCS and 2D reads have the same bias of homopolymer pattern to specific bases: A and T in insertions and G and C in deletions. Moreover, insertions of G and C have a “tight homopolymer pattern”: both upstream and downstream bases are the same as the inserted bases (“diagonal spots” in Figure 3 and Supplementary Figure S4). Overall, the homopolymer pattern of errors is more pronounced in the raw PacBio data (subreads), but not very clear in the raw ONT data (1D reads). Regardless of the difference in sequencing platform, the overall error patterns of CCS and 2D both contain homopolymer indels, which may be due to the consensus sequence algorithm. The specific mismatches of ONT data may be caused by some difficult case contexts for the basecaller.

In spite of the higher overall error rate, the error pattern of the PacBio C2 data is almost the same as P6-C4 data, while the C2 CCS reads have a “loose” rather than the “tight” homopolymer pattern of P6-C4 data for indels ( Supplementary Figure S4). Compared to ONT R9 data, the error patterns of R7 data (both 2D and 1D reads) are mosaic, with a few predominant errors ( Supplementary Figure S4). Only the “tight homopolymer pattern” of indels is observed in R7 2D reads. Therefore, PacBio and ONT data have been improved dramatically, except for some systematic errors at homopolymers and specific contexts.

### Isoform identification in SIRVs by Illumina, PacBio and ONT

Our next goal was to investigate the advantages of PacBio and ONT long reads for transcriptome analysis over Illumina short reads. We first compared the performance of gene isoform identification using the gold standard Spike-In RNA Variant Control mixes (SIRVs), which contain 68 isoforms of 7 genes with various splicing complexity and known abundance. This allows the evaluation of isoform recall by PacBio, ONT and Illumina data. We reconstructed isoforms from Illumina short reads by the reference-guided mode of StringTie ^41^ with three types of SIRV annotation libraries: the “correct” library containing all 68 truly-expressed isoforms, the “insufficient” library containing 43 of 68 truly-expressed isoforms, and the “over-annotated” library containing 68 truly-expressed isoforms and 32 additional unexpressed isoforms (see Methods). None were able to report all 68 truly expressed isoforms (44, 63 and 62, respectively; Table 2). When the reconstruction was guided by the "insufficient" SIRV annotation library, only 20.00% (5 of 25) of missing isoforms were rescued, along with 33 false positive predictions. When guided by the “over-annotated” SIRV annotation library, 46.87% (15 of 32) of unexpressed, but annotated, isoforms were incorrectly reported, with an additional 24 false positive predictions. Even if the assembly was guided by the "correct" SIRV annotation library, which is rarely available in practical transcriptome analysis, short reads identified 92.65% (63 of 68) annotated isoforms, but with 27 false positive predictions. These results demonstrated the incompleteness or high false positive rate of isoform reconstruction by short reads. In contrast, ONT directly detected all 68 expressed isoforms, and PacBio missed only one, isoform SIRV618, which is 219 bp and may be filtered out by size selection in PacBio library preparation. Thus, PacBio and ONT long reads show a far superior performance in isoform identification over short reads.

**Table 2.** Performance of Illumina, PacBio and ONT on isoform identification in the gold standard SIRVs.

Strategy (SIRV annotation library)	True positive	False positive
Illumina (with “insufficient” SIRV annotation library)	39 + 5 *	33
Illumina (with “correct” SIRV annotation library)	63	27
Illumina (with “over-annotated” SIRV annotation library)	62	24+15 **
PacBio (with “correct” SIRV annotation library)	67	-
ONT (with “correct” SIRV annotation library)	68	-

*In the “insufficient” SIRV annotation library, 25 isoforms are not included but are truly-expressed. Of these 25 isoforms, 5 isoforms were rescued when using Illumina short reads data. **In the “over-annotated” SIRV annotation library, 32 isoforms are included but are not truly-expressed. Of these 32 isoforms, 15 isoforms were assembled.

### Isoform identification in hESCs by PacBio, ONT and Hybrid-Seq

**Figure 4.**
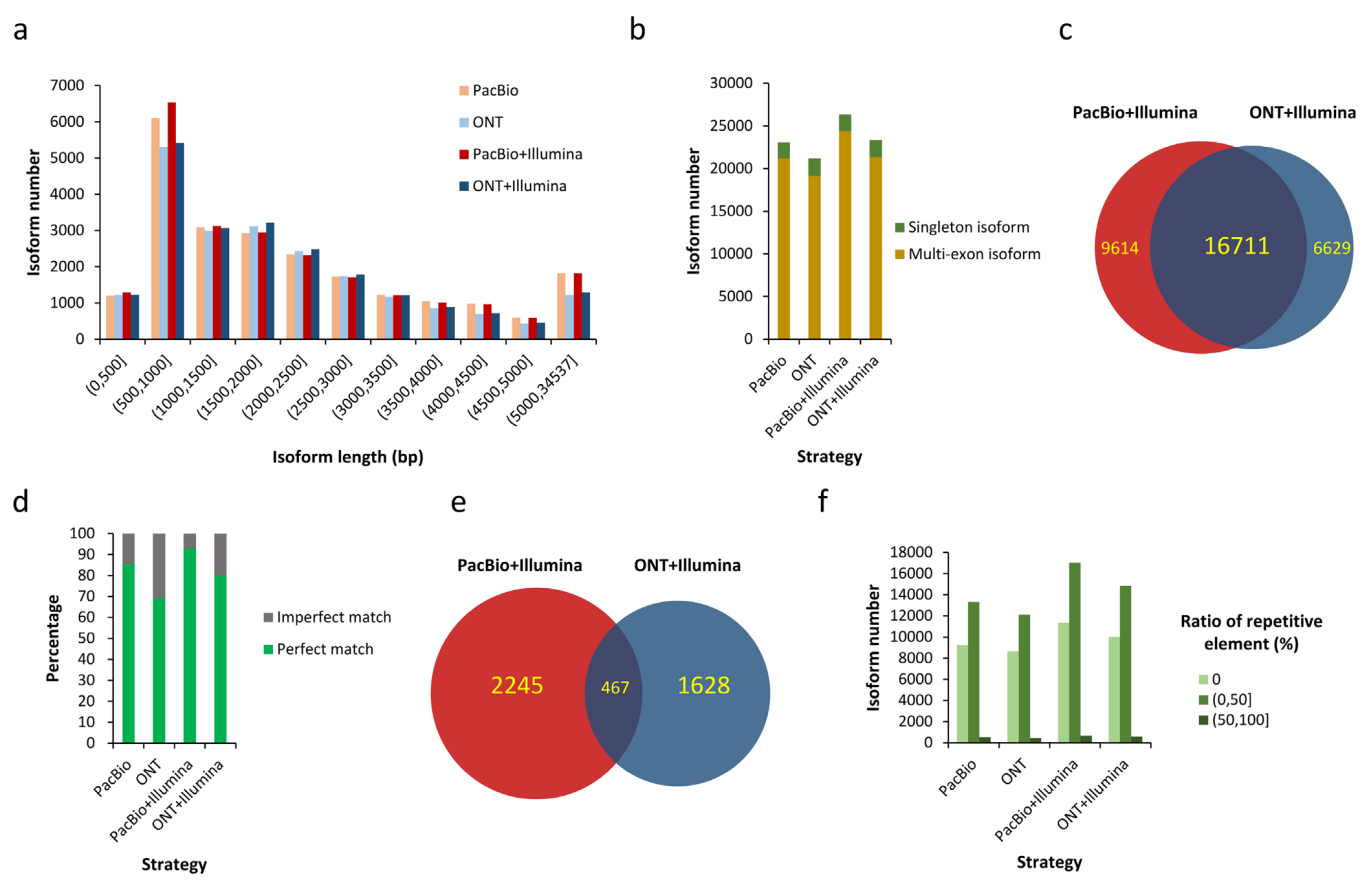
Isoform identification in human embryonic stem cells. ( **a**) Length distribution of isoforms identified by full-length by long read only and Hybrid-Seq strategies. ( **b**) Numbers of identified isoforms with single exon (singleton isoform) and multiple exons (multi-exon isoform). ( **c**) Overlap between isoforms identified by two Hybrid-Seq strategies. ( **d**) Accuracy of splice sites detected by four strategies. Perfect means the detected splice sites exactly match known splice sites annotated by Gencode (version 24). Imperfect means the detected splice sites are shorter or longer than known splice sites annotated by Gencode (version 24). ( **e**) Overlap between novel isoforms identified by two Hybrid-Seq strategies. ( **f**) Numbers of identified isoforms with different ratios of repetitive elements. ONT: Oxford Nanopore Technologies; PacBio: Pacific Biosciences.

We further evaluated the performance of PacBio and ONT in identifying isoforms from hESCs (H1 cell line, see Methods). In total, 919,158 mappable PacBio reads and 923,671 mappable ONT reads were used. A total of 57,868 and 59,098 Gencode-annotated isoforms were detected by PacBio and ONT reads, including 23,067 and 21,196 full-length isoform detection, respectively ( Figure 4A and Supplementary Figure S5). The full-length isoform identification rates were 47.14% and 44.79%, respectively. For the >1kb isoforms that are difficult to detect by short reads, PacBio and ONT directly detected 15,764 and 14,669 full length transcripts ( Figure 4A). Thus, ONT shows comparable sensitivity with PacBio for full-length isoform detection.

Next, we identified isoforms from two Hybrid-Seq datasets: PacBio+Illumina and ONT+Illumina. Firstly, the long reads were corrected by LSC (version 1 beta) ^18^ and Illumina reads, and the number of mappable reads increased to 951,258 and 933,762 for PacBio and ONT, respectively (see Methods). Furthermore, error correction greatly improved overall error rates and context-specific errors patterns ( Supplementary Figure S4). By inputting the corrected long reads and Illumina reads to IDP, 26,325 and 23,340 Gencode-annotated isoforms were identified by full length by PacBio+Illumina and ONT+Illumina, respectively ( Figure 4A), demonstrating the superior sensitivity of Hybrid-Seq over long reads only to identify isoforms. For multi-exon isoforms that are difficult to be constructed by short reads alone, the full-length isoform identification ratios were as high as 92.82% and 91.48% for PacBio+Illumina and ONT+Illumina, respectively ( Figure 4B). Whereas 16,711 isoforms were identified by both Hybrid-Seq datasets, the overlap ratios of identified isoforms were not very high (PacBio+Illumina: 63.48% and ONT+Illumina: 71.60%; Figure 4C). That is, the two Hybrid-Seq datasets rescued significant numbers of isoforms that were missed by the other (9,614 and 6,629 for PacBio+Illumina and ONT+Illumina, respectively). These discordant isoforms were mostly multi-exon isoforms ( Supplementary Figure S6).

Imperfect alignments of error-prone long reads subsequently result in ambiguous determination of splice sites/exon boundaries within the multi-exon isoforms. Using splice sites annotated by the reference library and or detected by short reads as the gold standard, 14.72% and 30.82% splice sites were incorrectly identified by PacBio and ONT, respectively ( Figure 4D and Supplementary Figure S7). By contrast, by correcting long reads with short reads and integrating short reads in isoform identification (i.e., by the tool IDP), the incorrectly identified rates were decreased to 7.05% and 19.94% for PacBio+Illumina and ONT+Illumina, respectively. Thus, Hybrid-Seq provides a higher resolution of the exon-intron structures within each identified isoform. In addition, PacBio showed a better performance of splice site determination for both long read only and Hybrid-Seq strategies, which is consistent with the lower error rates than ONT.

With the determination of high-resolution exon-intron structure and the consistent evidence from both TGS and SGS data, we can discover and annotate significant amounts of novel multi-exon isoforms accurately: 2,712 and 2,095 by PacBio+Illumina and ONT+Illumina, respectively ( Figure 4E). Compared with the overlap of annotated isoform detection ( Figure 4C), only a minority of novel isoforms (467) were identified by both Hybrid-Seq strategies ( Figure 4E). Besides the possible technological difference, the distinct coverage of novel isoforms by our PacBio and ONT data may be attributable to sampling differences.

We also illustrated the utility of long reads to identify isoforms with repetitive elements (see Methods). Approximately 60% of isoforms identified by PacBio (13,830; 59.96%), ONT (12,559; 59.25%), PacBio+Illumina (17,672; 60.86%) and ONT+Illumina (15,426; 60.65%) contained repetitive elements, and in particular, a significant amount of isoforms identified contained > 50% repetitive elements (516, 451, 665 and 593, respectively; Figure 4F). Reconstruction of isoforms with repetitive elements is difficult for short reads ^43^, while it is relatively easily and accurately accomplished using long reads.

### Isoform abundance estimation by PacBio, ONT and Hybrid-Seq

**Figure 5.**
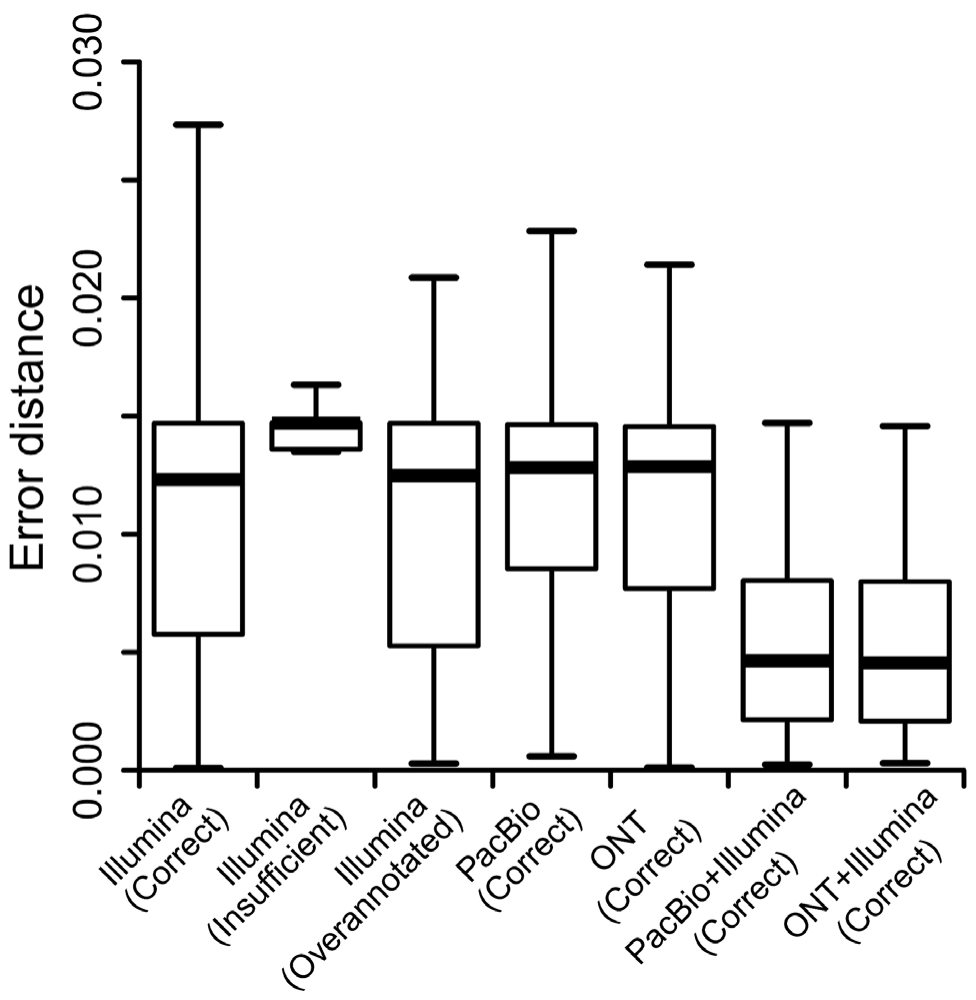
Estimation errors of isoform abundance estimation in Spike-in RNA Variant data. The X axis shows 7 strategies. The label “correct”, “insufficient” and “over-annotated” in parentheses represent three different SIRV annotation libraries, respectively. The Y axis shows the euclidean distance between real relative expression percentage (1/68≈0.15) and estimated relative expression percentage (for more details see Methods). ONT: Oxford Nanopore Technologies; PacBio: Pacific Biosciences.

We evaluated the performance of PacBio, ONT, Hybrid-Seq and Illumina data on isoform quantification, using the gold standard SIRVs (see Methods). The abundance of all 68 SIRVs are the same and here we evaluated the estimation of their uniform relative abundance (1/68≈0.15). We first tested Illumina data, with the isoform library reconstructions guided by the three aforementioned SIRV annotation libraries. The median estimation errors were 0.12, 0.18 and 0.12 for “correct”, “insufficient” and “over-annotated” annotation libraries, respectively ( Figure 5). It suggests isoform abundance estimation is less accurate when expressed, but unannotated isoforms are missed in isoform identification (e.g. the “insufficient” library). In contrast, when isoforms were identified and quantified by Hybrid-Seq, the median estimation errors were as low as 0.06 for PacBio+Illumina and 0.05 for ONT+Illumina. Additionally, we also observed high median estimation errors when using long reads only (0.15 for PacBio and 0.13 for ONT). This reflects the drawbacks of TGS long reads in quantitative analysis, such as low throughput and bias, yet a better isoform library can be obtained than with short reads only. Overall, although the errors from all estimation methods are of the same order of magnitude of the relative abundance (0.15), Hybrid-Seq provides a better strategy to fully utilize PacBio and ONT long reads in transcriptome analysis.

### Complexity of the hESC transcriptome

Alternative splicing and alternative polyadenylation, produce a substantial number of isoforms with different lengths, exon usage and polyadenylation sites, which greatly enriches the complexity of the human transcriptome ^44– 46^. The average lengths of identified isoforms were 1,759bp, 1,670bp, 1,848bp and 1,747bp for PacBio, ONT, PacBio+Illumina and ONT+Illumina, respectively ( Supplementary Figure S8). The longest isoform (Gencode ID: ENST00000262160.10), which was simultaneously identified by all four strategies, was 34,537bp.

For multi-exon isoforms, an average of ~8 exons in each isoform was identified by each of the four strategies ( Supplementary Figure S9). However, the largest numbers of exons contained within single isoforms differed among PacBio, ONT, PacBio+Illumina and ONT+Illumina datasets: 64, 49, 67 and 52, respectively. When considering the isoforms with ≥30 exons, both PacBio (243) and PacBio+Illumina (367) were capable of identifying more isoforms than ONT (84) and ONT+Illumina (169). These results indicate both technologies can identify isoforms with many exons, but there is not sufficient evidence to reveal conclusive difference between the sequencing platforms. PacBio being size-selected and ONT’s lack of size-selection may also have contributed to the observed differences.

**Figure 6.**
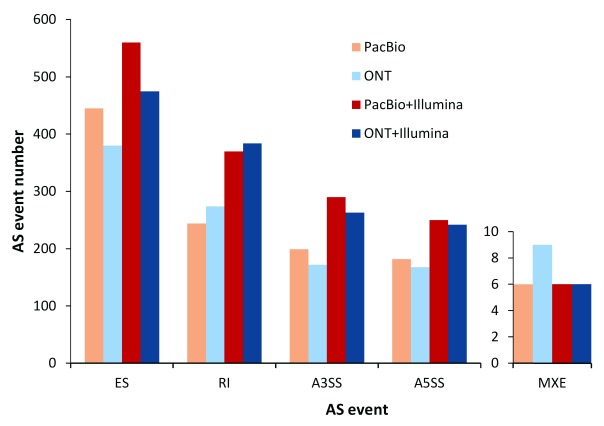
Numbers of different alternative splicing (AS) events in human embryonic stem cells transcriptome. A5SS: alternative 5’ splicing site; A3SS: alternative 3’ splicing site; ES: exon skipping; RI: retained intron; MXE: mutually exclusive exons; ONT: Oxford Nanopore Technologies; PacBio: Pacific Biosciences.

Alternative splicing events lead to the diversity of isoform expression. PacBio, ONT, PacBio+Illumina and ONT+Illumina identified 1,076, 1,003, 1,476 and 1,370 alternative splicing events, respectively ( Figure 6). On average, the most frequent alternative splicing events identified were exon skipping (37.96%), followed by intron retentions (25.77%), alternative 3’ splicing sites (18.62%) and alternative 5’ splicing sites (17.07%). A few mutually exclusive exons events (0.57%) were also discovered.

As reported recently, PacBio data can identify alternative polyadenylation sites ^23^. In our data, poly(A/T) tails were detected in 76.71% PacBio CCS reads and 59.75% ONT 2D reads. It shows the comparable potential of ONT to identify alternative polyadenylation sites as PacBio.

### Functional analysis of identified isoforms in hESCs

**Figure 7.**
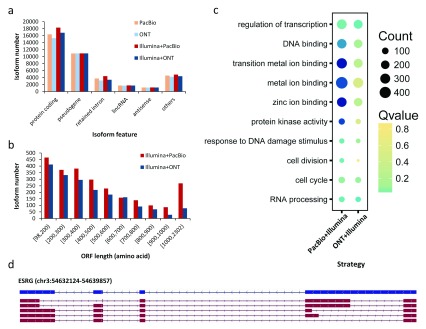
Functional analysis of identified isoforms. ( **a**) Feature statistics of isoforms annotated by Gencode (version 24). ( **b**) Length distribution of open reading frames (ORFs) of novel isoforms identified by two Hybrid-Seq strategies. ( **c**) Gene enrichment analysis of genes with at least one novel isoform identified by two Hybrid-Seq strategies. ( **d**) Five novel isoforms (red tracks) of the human embryonic stem cell-relevant gene *ESRG* were identified by two Hybrid-Seq strategies. The topmost isoform (blue track) is annotated by Gencode (version 24). ESRG: Embryonic Stem Cell Related Gene; ONT: Oxford Nanopore Technologies; PacBio: Pacific Biosciences.

For the Gencode-annotated isoforms identified by PacBio, ONT, PacBio+Illumina and ONT+Illumina, 42.51%, 41.87%, 44.06% and 43.78% were protein-coding, respectively ( Figure 7A) and the ratios of pseudogenes were 28.38%, 29.99%, 26.38% and 28.46%. Some isoforms were annotated as retained introns (9.48%, average), lincRNA (4.47%, average) and antisense transcripts (3.02%, average).

For novel isoforms identified by Hybrid-Seq, we evaluated the protein coding potential by GeneMarkS-T (see Methods). Open reading frames (ORFs) with >97 amino acids were found in 92.59% (2,511/2,712) and 89.40% (1,873/2,095) novel isoforms identified by PacBio+Illumina and ONT+Illumina, respectively, with average lengths of 516 and 427 amino acids ( Figure 7B). The longest ORFs were 2,302 and 1,980 amino acids, respectively.

We performed gene enrichment analysis for those genes with ≥1 novel isoform. Most genes were enriched in transcription regulation, DNA binding and metal ion binding processes ( Figure 7C), which are likely important for human embryonic development. Some other enriched genes have protein kinase activity and are associated with DNA damage response, cell division, cell cycle and RNA processing processes.

Furthermore, 26 hESC-relevant genes expressed ≥1 novel isoform, which was supported by PacBio or ONT full length data ( Supplementary Table 3). For example, five novel isoforms (red track in Figure 7D) were full-length identified by both PacBio and ONT long reads in *ESRG* (Embryonic Stem Cell Related Gene), which is required for maintenance of human embryonic stem cell pluripotency ^47^. These isoforms were not annotated by the existing annotation libraries (Gencode, Ensembl or RefSeq) and contained alternative 5’ splicing sites and alternative 3’ splicing sites.

## Discussion

Overall, PacBio and ONT are similar: long read length, high error rate and relatively low throughput. However, they have distinct aspects, such as homopolymer error in PacBio and context-specific mismatches in ONT. PacBio sequences a molecule multiple times to generate high-quality consensus data, while ONT can only sequence a molecule twice. Together with the higher quality of the raw data, PacBio can generate extremely-low-error-rate data for high-resolution studies, which is not feasible for ONT. PacBio has better data quality for most aspects, such as error rate and mappability, especially for the consensus data (CCS vs. 2D). However, ONT has a few advantages: in addition to slightly longer mappable length, ONT MinION provides very high throughput as the nanopores can sequence multiple molecules. The cost for our ONT data generation was 1,000–2,000USD. Since sequencing cost is a significant obstacle of TGS application, the relatively high throughput and affordability makes ONT promising for many applications, especially for genome-wide and transcriptome-wide studies, requiring large amounts of data.

With a comprehensive understanding of the data features of PacBio and ONT, we can perform better data analysis and bioinformatics method development. We found a significant number of chimeric reads, which may be generated by either library preparation artifacts or failure of removing adaptors. Thus, it is important to filter these problematic long reads before further analyzing TGS data. However, we cannot filter the data using a simple cutoff: though the subreads and 1D reads are not as accurate as CCS and 2D reads, they are useful because of their reasonable mappability. In particular, error correction by short reads can improve the error rates and increase the mappability. The subreads and 1D reads consist of ~50–60% of the total data provided from the machines, and moreover, many ONT “fail” reads are also mappable, though they are often discarded. Therefore, sophisticated data analysis and bioinformatics methods, such as error correction, are required to rescue or to make better use of these data. The specific error pattern lays the groundwork for better method development. Similarly, the studies of error pattern can also benefit the development and applications of both long-reads only and Hybrid-Seq approaches for nucleotide analysis, such as SNP calling. We notice that our results are subjected to a compound workflow, including library preparation, sequencing, base calling, and analysis software. However, as we used standard protocols/analyses, these results can still serve as an informative reference.

In fact, studies concerning ONT have recently validated its utility in genome assembly ^48^. For transcriptome analysis, we demonstrated the capability of both ONT and PacBio to provide precise and complete isoform identification of a small gold standard library SIRVs. For complicated transcriptomes (e.g., hESCs), ONT also provided comparable results to PacBio. However, with the higher data quality, PacBio has a slightly better overall performance, such as discovery of transcriptome complexity and sensitive identification of isoforms. Furthermore, we successfully improved the overall transcriptome analysis by ONT+Illumina, which is the first study to use ONT data in the Hybrid-Seq strategy. This similar improvement is also observed in PacBio Hybrid-Seq over PacBio alone, as reported previously ^11^, because short reads not only correct the errors of long reads, but also improve abundance estimation and splice site determination. Abundance estimation could be also benefit from a more precise isoform library by Hybrid-Seq. In addition, the requirement of consistency between TGS and SGS data could also filter out many false positives, such as false gene fusion detection from library preparation artifacts. It is notable that PacBio and ONT have their unique discoveries missed by each other, such as novel isoforms.

Additionally, we established that the technology improvements from the previous to the latest sequencing models of both PacBio and ONT are significant, including error rates and yields (especially for ONT). Therefore, the applications of both PacBio and ONT are expected to increase dramatically in the near future, and the results and the comparisons above provide a reference for analyzing PacBio and ONT data. This study also provides an informative paradigm for the application of PacBio and ONT to analyze transcriptomes by long reads only and their corresponding Hybrid-Seq strategies.

## Software and data availability

The AlignQC software described herein is freely available for use and can be downloaded from: http://www.healthcare.uiowa.edu/labs/au/AlignQC/.

Source code available from: https://github.com/jason-weirather/AlignQC

Archived source code as at time of publication: doi, 10.5281/zenodo.224125 ^49^ ( https://zenodo.org/record/224125#.WHUFN1WLTcs)

License: Apache 2.0

Reference sequence and annotation versions are described in Supplementary Table 1.

## Supplementary material

**Supplementary Figures 1–9 (in zipped file).**

**Figure S1. Read length distribution per-cell.** The read length distribution per Pacific Biosciences (PacBio) Single Molecule Real Time (SMRT) cells and Oxford Nanopore Technologies (ONT) flow cell are shown. Read counts are plotted from bins of 200bp. Panels represent each type of read generated by the platforms: circular consensus sequence (CCS) and subreads for PacBio, and for ONT there are 2D, 1D template and 1D complement. ONT reads classified as pass or fail are plotted as different colors. Note* that PacBio C2 subreads are plotted on a different y-axis scale than PacBio P6-C4 for visibility of the lower per-cell throughput from the older C2 chemistry. The number of cells (n) and number of reads ( **r**) for each technology is listed at the top of each panel.

**Figure S2.1. Length distribution of reads and mappability**. The length distribution of alignments and their mapped portions shown for Oxford Nanopore Technologies (ONT) 2D, and 1D (template strand) reads and Pacific Biosciences (PacBio) circular consensus sequence (CCS) and subreads in the main text are supplemented here with additional read sets of interest. High quality reads represent ONT 2D reads and PacBio CCS reads. The Raw reads represent ONT template strand reads and PacBio subreads. The 1D complement strand of ONT is now included. Columns have also been added for side-by-side comparison with LSC-corrected reads. Rows contain results for both current PacBio P6-C4 and ONT R9 along with older PacBio C2 and ONT R7 chemistries.

**Figure S2.2. Length distribution of reads and mappability for ONT ‘fail’**. These results show the mappability of ‘fail’ classified ONT reads.

**Figure S3. Mismatch error pattern regardless of platform.** A ‘C’ followed by a ‘G’ followed by any base or the reverse complement that sequence is a pattern observed cross the low error rate Illumina and is also observed even when reads perfectly match the reference sequence. PacBio: Pacific Biosciences.

**Figure S4. Pacific Biosciences (PacBio) and Oxford Nanopore Technologies (ONT) context-specific errors.** Along with the context-specific errors plotted in the main text, this plot adds side-by-side comparisons of LSC-corrected data reads. Each error type (‘insertion’, ‘deletion’, and ‘mismatch’) for each result is individually scaled for better resolution of errors present in each result.

**Figure S5. Length statistics of ‘partial’ isoforms detected by four strategies.** ONT: Oxford Nanopore Technologies; PacBio: Pacific Biosciences.

**Figure S6. Overlap between isoforms identified by two Hybrid-Seq strategies.** ( **a**) Overlap between multi-exon isoforms. ( **b**) Overlap between singleton isoforms. ONT: Oxford Nanopore Technologies; PacBio: Pacific Biosciences.

**Figure S7. Statistics of splice site accuracy.** This figure does not include the perfectly matched splice sites (relative distance is equal to 0). The negative values and positive values represent the truncated (shorter than known splice sites) and elongated (longer than known splice sites) nucleotide distance from the reference splice site, respectively. ONT: Oxford Nanopore Technologies; PacBio: Pacific Biosciences.

**Supplementary Figure S8. Length statistics of identified isoforms.** ONT: Oxford Nanopore Technologies; PacBio: Pacific Biosciences.

**Supplementary Figure S9. Exon number statistics of identified isoforms.** ONT: Oxford Nanopore Technologies; PacBio: Pacific Biosciences.

**Supplementary Table 1. Reference sequences and annotations.** The source, address, version, and accession numbers are provided, when available, for reference sequences and annotations.

**Supplementary Table 2. Summary statistics comparing technologies.** The statistics of reads outputted by each technology are organized by row. The colored columns, A–F, represent the subset of the technology being shown. These variables include the Platform (A), Chsemistry (B), and Correction status (C). GeneralType (D) describes whether reads are high quality (HQ) consensus sequences, the raw nucleotides (possibly multiple per molecule), or the single-best aligned sequence representing a single molecule. The ReadType (E) more specifically defines the GeneralType based on the platform-specific outputs. Finally column (F) specifies whether reads were called as ‘pass’ or ‘fail’ for the Oxford Nanopore Technologies (ONT) platform. The remaining columns provide yield, length, mappability, and error rate information.

**Supplementary Table 3. Novel human embryonic stem cell (hESC)-relevant isoforms.** The novel isoforms of 22 hESC-relevant genes are shown along with a functional description, the number of supporting full-length Pacific Biosciences (PacBio) and Oxford Nanopore Technologies (ONT) long reads. Mapping information to the hg38 genome is also provided.
