# Supplementary material for: Comprehensive comparison of Pacific Biosciences and Oxford Nanopore Technologies and their applications to transcriptome analysis
Source: F1000Res. 2017 Jun 19;6:100. Originally published 2017 Feb 3. [Version 2] doi: 10.12688/f1000research.10571.2 (PMC5553090; doi:10.12688/f1000research.10571.2)
Supplement: Peer review history

# Peer review: Referee response for version 2

**DOI:** 10.5256/f1000research.12836.r23594

**Published:** 2017-08-04

**Authors:** Jingyi Jessica Li

**Affiliations:** 1 Department of Statistics, University of California, Los Angeles, Los Angeles, CA, USA

The authors have done a careful revision to successfully address all my questions about the previous version.

I have read this submission. I believe that I have an appropriate level of expertise to confirm that it is of an acceptable scientific standard.

# Peer review: Referee response for version 2

**DOI:** 10.5256/f1000research.12836.r23595

**Published:** 2017-07-31

**Authors:** Hagen Tilgner

**Affiliations:** 1 Weill Cornell Medical College , New York City, NY, USA

Weirather and coworkers have much improved their manuscript. Specifically the rewritten version of the abstract has lowered the probability of misleading readers drastically. There are still three small issues that I’d ask the authors to take care of. Since they are straight-forward, I think the authors can take care of this without further review. So, I’ll say congratulations (from my side).

 Point 1 (I touched on that in my first review): Regarding Figure 5: Figure 5’s legend says “the Y axis shows the euclidean distance between real relative expression percentage and estimated relative expression percentage”. Therefore I expect one single value for each dataset (the euclidean distance taking into account multiple isoforms). But I see a boxplot (representing of course multiple values).

 Please clarify what the different data points in each boxplot are. The seven genes? Different subsamples of reads? Something else? I bet that adding one or two phrases to the legend of figure 5 will make it obvious, for readers who were not involved in the research.

 Point 2: The text and the legend of Figure 5 says “(1/68≈0.15)”, which is off by an order of magnitude.

 Point 3 (I touched on that in my first review): In the section about alternative splicing quantification (Figure 6), the 10% cutoff, that I asked the authors to use appears to have changed the results, so that they are consistent with my expectations. Thus, this cutoff should be mentioned for reproducibility (apologies if I overlooked it) and the two publications I mentioned in my previous review cited.

 Again, I do not think this requires re-review.

I have read this submission. I believe that I have an appropriate level of expertise to confirm that it is of an acceptable scientific standard.

# Peer review: Referee response for version 1

**DOI:** 10.5256/f1000research.11392.r20639

**Published:** 2017-03-01

**Authors:** Hagen Tilgner

**Affiliations:** 1 Weill Cornell Medical College , New York City, NY, USA

The manuscript by Weirather and coworkers compares two third generation sequencing protocols (Pacific Biosciences – PacBio as well as Oxford Nanopore technologies – ONT) in terms of their performance for RNA sequencing. It concludes that both technologies can be used for transcriptome analysis with PacBio having advantages in terms of sequencing errors and consequently alignability while ONT gives higher sequencing throughput.

 Generally speaking, this is an important topic, which many readers will find of interest. The manuscript has a lot of very informative information that can guide researchers in their experiments.

 On the flipside there are also a couple of instances where readers might be misled if they are not specialists in the field. I will detail these points of the manuscript below and what I think should be done in order to address them. The authors should be able to address these issues without many difficulties. This will then be an important contribution to the field.

**Strengths:**

 1) The demonstration of the dependence of sequencing quality (or the Fraction of read aligned) on read length (figure 2) both for single pass reads (subreads for PacBio and 1D for ONT) and for multi-pass consensus reads (CCS for PacBio and 2D reads for ONT) is very useful. Future readers will be able to have a good estimate of what they might expect for their genes of interest.

 2) The comparison of the type of error (figure 3) is very useful.

 3) Likewise the chimera analysis is useful to understand the limitations one must be aware of when planning experiments.

**Weaknesses and solutions and other questions:**

 1a) The first drawback is that the experiment for PacBio and ONT is not exactly identical. PacBio libraries underwent size selection, whereas ONT libraries did not (as the authors indicate in an upfront way), although in theory, I do not see why this could not have been done for ONT. The reason, I would guess, is that for ONT size fractions are not required (just as they were not in our 2015 synthetic long read isoform paper ^1^). Nevertheless, this leaves us with the problem that we cannot exactly understand what are characteristic differences between ONT and PacBio and what may be linked to size selection. I think the authors should indicate in a prominent place (e.g. the abstract) that this is a comparison of a “PacBio experiment using size fractions” and a non-size-selected Oxford Nanopore experiment. This is of importance because many readers may only read the abstract and look at the figures – and the current version could cause them to miss this point.

 1b) From the above drawback, it follows that for PacBio the authors need to choose how much sequencing is devoted to the four size bins (1,4,4 and 3 SMRT cells, I believe are chosen) but for ONT this is not done. Therefore the length profile in figure 1 (top) is a function of the Oxford Nanopore system and the cDNA sample only, but the distributions (bottom) for PacBio also depend on the employed size selections and SMRT cell numbers. In principle one could (if one wanted to) make the 500bp-1kb bin the most prominent bin in the PacBio length distribution, by also using 4 SMRT cells for this bin. Conversely one could give more weight to other bins. On the upside, this means one can zoom in on sizes of interest. On the downside, one must carefully consider the implications for the transcriptome of interest. The important point here is, again, that all of this could also have been done for ONT. I suggest to make readers aware of this in an obvious way in the legend of figure 1.

 1c) Additionally, in figure 2, I would remove the leftmost boxplot for each panel (the overall Fraction of Read aligned), because in the case of PacBio this would change, if one were to use different amounts of sequencing for different bins (because these bins differ, as the authors show, in terms of alignability). The “Fraction of Read Aligned” broken up by length bins however is highly informative. Please do keep this by all means!

 2a) Regarding isoform abundance estimation from SIRVs (figure5): The authors employed the E0 mix of the SIRVs, in which all different isoforms are of equal abundance. This is very different from real-world situations, in which different genes but also different isoforms from the same gene can be of very different expression level. The authors note earlier that ONT has advantages in sequencing depth (at the cost of quality), which (we would hope) would lead to better isoform quantification for lowly expressed genes and minor isoforms– but using the E0 mix we cannot tell (while we could have, I think with the E1 mix). Reading the paper, I was searching for the use of the E1 and E2 mixes which could have answered these questions. It would be good to point out that lowly expressed gene and minor isoform quantification were not addressed here.

 2b) Also, regarding the isoform abundance estimation, my first impression was “these are actually very small errors” when looking at the y-axis of figure 5. My current understanding of the situation is however different: As the authors point out, the actual expression of each isoform is 1/68~=0.015, meaning that the errors are of the same order of magnitude as the (uniformly) expressed transcripts – and a bit less for error corrected reads. If my reading of the situation is accurate, then this should be noted somewhere.

 3) Other points:

- Page 8 left column: fig 2 is referenced for “ONT data have particularly higher trans-chimeric rates in very long reads (>4kb)”. Shouldn’t this be fig. 1 ?
- Page 10, right column, end of first paragraph: when referring to table 2, it is not obvious (apologies, if I missed it) what kind of long-reads (ONT-1D vs. ONT-2D vs. ONT-errorCorrection and PacBio-CCS vs. PacBio-subread vs. PacBio-errorCorrected) are used. Earlier parts of the paper use abbreviations like ONT-1D or PacBio-subreads, but not here.
- A similar statement is true for figure 5 and the corresponding text (“when using long reads only”): it is not clear if PacBio-CCS or PacBio-subreads are used (and the same for ONT) when comparing to the error-corrected subreads.
- For figure 5, it is somewhat difficult to understand, what was exactly done. The authors say that, they used the “Euclidean distance” between REP and estimated REP. The way I understand it, is that the authors calculated REP and estimated REP for each transcript, and then calculated the Euclidean distance for each isoform. In this case (one dimension only) the Euclidean distance reduces to the absolute value of REP minus estimated REP. If this was done, this simpler way of saying it, is advantageous, I believe. Using the word “Euclidean distance” makes me expect multidimensionality. This would suggest that the authors have a vector of isoform expression values for each gene (or maybe multiple samples)? That would imply that the boxplots only represent 7 dots for the 7 SIRV genes…please clarify so that there is no doubt.
- The section “Isoform Identification in hESCs by PacBio, ONT and Hybrid-Seq” is difficult to read. This may stem from the terms “full length rates” and “full-length isoform identification rates”. It is not fully clear, if they mean the same or different things; What is exactly meant? Is it “fraction of discovered annotated isoforms that are seen at least once in a full length read” or “fraction of reads that are judged as full-length” or something else? Please clarify.
- Page 12, the third paragraph, regarding the discovery of isoforms with >=30 exons. The correct finding of isoforms with lots of exons of course depends on error-rate (which is linked to getting all splice sites correctly) and having long enough reads. In the absence of a size selection experiment for the Minion, one cannot prove that the observed difference between PacBio and Minion would not be rendered smaller (probably not totally removed though – because of the higher Minion error rate), with a size selection experiment for the Minion. I would mention that.
- Regarding the quantification of alternative splicing events … there are many publications that suggested exon skipping is the most frequent type of alternative splicing in humans. There are reports that have reported high occurrence of intron retention – but to my knowledge not that intron retention is more frequent than exon skipping. For example Braunschweig et al, Genome Research 2014 ^2^ using short reads and our own paper Tilgner et al, Nature Biotech, 2015 ^1^ using long reads. The authors could also use a minimum frequency of each single alternative event (e.g. 10% as in the papers referenced above) to distinguish splicing errors and few intermediate RNA molecules from “real” isoforms. This may change the relative abundance of each type of splicing event.
- In the last paragraph on page 12, the word “alterative” is used. I assume this should be “alternative”. If this is a spelling mistake, there may be more.

I have read this submission. I believe that I have an appropriate level of expertise to confirm that it is of an acceptable scientific standard, however I have significant reservations, as outlined above.

## References

[1] TilgnerHJahanbaniFBlauwkampTMoshrefiAJaegerEChenFHarelIBustamanteCDRasmussenMSnyderMP: Comprehensive transcriptome analysis using synthetic long-read sequencing reveals molecular co-association of distant splicing events. *Nat Biotechnol*.2015;33(7) : 10.1038/nbt.3242 736-42 10.1038/nbt.3242 25985263PMC4832928

[2] BraunschweigUBarbosa-MoraisNLPanQNachmanENAlipanahiBGonatopoulos-PournatzisTFreyBIrimiaMBlencoweBJ: Widespread intron retention in mammals functionally tunes transcriptomes. *Genome Res*.2014;24(11) : 10.1101/gr.177790.114 1774-86 10.1101/gr.177790.114 25258385PMC4216919

# Response

**Published:** 2017-06-07

**Authors:** Kin Fai Au

**Affiliations:** University of Iowa, USA

**We greatly appreciate your time and thoughtful questions and critiques of our manuscript “Comprehensive comparison of PacBio and Oxford Nanopore Technologies and their applications to transcriptome analysis.”  These are addressed in this point by point response and in the corresponding manuscript revisions.**

*HT:  The manuscript by Weirather and coworkers compares two third generation sequencing protocols (Pacific Biosciences – PacBio as well as Oxford Nanopore technologies – ONT) in terms of their performance for RNA sequencing. It concludes that both technologies can be used for transcriptome analysis with PacBio having advantages in terms of sequencing errors and consequently alignability while ONT gives higher sequencing throughput.*

*Generally speaking, this is an important topic, which many readers will find of interest. The manuscript has a lot of very informative information that can guide researchers in their experiments.*

*On the flipside there are also a couple of instances where readers might be misled if they are not specialists in the field. I will detail these points of the manuscript below and what I think should be done in order to address them. The authors should be able to address these issues without many difficulties. This will then be an important contribution to the field.*

**

***Strengths:***

**

*1) The demonstration of the dependence of sequencing quality (or the Fraction of read aligned) on read length (figure 2) both for single pass reads (subreads for PacBio and 1D for ONT) and for multi-pass consensus reads (CCS for PacBio and 2D reads for ONT) is very useful. Future readers will be able to have a good estimate of what they might expect for their genes of interest.*

**

*2) The comparison of the type of error (figure 3) is very useful.*

**

*3) Likewise the chimera analysis is useful to understand the limitations one must be aware of when planning experiments.*

**

***Weaknesses and solutions and other questions:***

**

*HT (1a):  The first drawback is that the experiment for PacBio and ONT is not exactly identical. PacBio libraries underwent size selection, whereas ONT libraries did not (as the authors indicate in an upfront way), although in theory, I do not see why this could not have been done for ONT. The reason, I would guess, is that for ONT size fractions are not required (just as they were not in our 2015 synthetic long read isoform paper* *^1^* *). Nevertheless, this leaves us with the problem that we cannot exactly understand what are characteristic differences between ONT and PacBio and what may be linked to size selection. I think the authors should indicate in a prominent place (e.g. the abstract) that this is a comparison of a “PacBio experiment using size fractions” and a non-size-selected Oxford Nanopore experiment. This is of importance because many readers may only read the abstract and look at the figures – and the current version could cause them to miss this point.*

**Thank you for strongly making this point.  The fact that PacBio was size selected and ONT was not deserves discussion and consideration. In fact, we did try size selection with ONT, but unfortunately it did not work in our hands and we haven't figured out the reason. Size selection is officially recommended for PacBio Iso-Seq protocol and has been validated by many published works, while there is so far no "official" protocol released by ONT. Therefore, transcriptome data collection without size selection was the only successful way that we could perform with ONT platform. We strongly encourage more follow-up studies to figure out an optimal protocol to generate transcriptome data with ONT platform.**

**Nevertheless, we agree size selection is a critical difference between the two sequencing data collections in our work and needs prominent mention in the manuscript.  To this end, we have modified the Abstract, Introduction, and first two figures to make specific mention of this difference.  We hope this change will make readers more clearly aware of this difference.**

*HT (1b):  From the above drawback, it follows that for PacBio the authors need to choose how much sequencing is devoted to the four size bins (1,4,4 and 3 SMRT cells, I believe are chosen) but for ONT this is not done. Therefore the length profile in figure 1 (top) is a function of the Oxford Nanopore system and the cDNA sample only, but the distributions (bottom) for PacBio also depend on the employed size selections and SMRT cell numbers. In principle one could (if one wanted to) make the 500bp-1kb bin the most prominent bin in the PacBio length distribution, by also using 4 SMRT cells for this bin. Conversely one could give more weight to other bins. On the upside, this means one can zoom in on sizes of interest. On the downside, one must carefully consider the implications for the transcriptome of interest. The important point here is, again, that all of this could also have been done for ONT. I suggest to make readers aware of this in an obvious way in the legend of figure 1.*

**We modified the legend of Figure 1 to point out the size selection step in PacBio data.  As mentioned above, we did not have a successful experiment doing size-selection of ONT or have an official protocol recommendation. To be clear, we do not want our lack of success in working size-selection into the ONT protocol to be misinterpreted as deficiency in the ONT platform.  Rather, we would prefer to defer topic of size selection in ONT until it has been better explored by ourselves or others in the community.**

*HT (1c):  Additionally, in figure 2, I would remove the leftmost boxplot for each panel (the overall Fraction of Read aligned), because in the case of PacBio this would change, if one were to use different amounts of sequencing for different bins (because these bins differ, as the authors show, in terms of alignability). The “Fraction of Read Aligned” broken up by length bins however is highly informative. Please do keep this by all means!*

**Thank you for this suggestion. While we agree that the most informative parts of the plot are the center and left panels, we feel the leftmost (all aligned reads) plot is somewhat useful for providing an overall view of alignability and would prefer to keep it.  In response to the other reviewer's comment, this plot was supplemented with the aligned read counts, which should improve the overall readability.**

*HT (2a):  Regarding isoform abundance estimation from SIRVs (figure5): The authors employed the E0 mix of the SIRVs, in which all different isoforms are of equal abundance. This is very different from real-world situations, in which different genes but also different isoforms from the same gene can be of very different expression level. The authors note earlier that ONT has advantages in sequencing depth (at the cost of quality), which (we would hope) would lead to better isoform quantification for lowly expressed genes and minor isoforms– but using the E0 mix we cannot tell (while we could have, I think with the E1 mix). Reading the paper, I was searching for the use of the E1 and E2 mixes which could have answered these questions. It would be good to point out that lowly expressed gene and minor isoform quantification were not addressed here.*

**Thank you for the suggestions.  We elected to use the E0 mix to have as many fixed variables as we possibly could to get a simple and clear readout on performance.  We aimed to evaluate how isoform identification and different types of sequencing coverage (by long reads or short reads) affect the isoform quantification. For example, hybrid sequencing strategies had better isoform identification by long reads (PacBio or ONT) and better quantitative information from short-read coverage (Illumina) in the statistical model, so they had better accuracy. We agree that including E1 and E2 is good to explore more issues in isoform quantification, such as the lowly-expressed ones. For example, lower sequencing coverage of lowly-expressed transcripts could contribute to the variance of abundance estimation. We could consider a separate manuscript to study all problems of isoform abundance estimation thoroughly.**

*HT (2b):  Also, regarding the isoform abundance estimation, my first impression was “these are actually very small errors” when looking at the y-axis of figure 5. My current understanding of the situation is however different: As the authors point out, the actual expression of each isoform is 1/68~=0.015, meaning that the errors are of the same order of magnitude as the (uniformly) expressed transcripts – and a bit less for error corrected reads. If my reading of the situation is accurate, then this should be noted somewhere.*

**We are sorry for the unclear description of Figure 5. We revised the section “Isoform abundance estimation by PacBio, ONT and Hybrid-Seq” and the legend of Figure 5 to clarify this issue.**

*HT (3):  Other points:*

*HT (3a):  Page 8 left column: fig 2 is referenced for “ONT data have particularly higher trans-chimeric rates in very long reads (>4kb)”. Shouldn’t this be fig. 1 ?*

**Yes, thank you so much for pointing this out. We have made this correction in the manuscript.**

*HT (3b):  Page 10, right column, end of first paragraph: when referring to table 2, it is not obvious (apologies, if I missed it) what kind of long-reads (ONT-1D vs. ONT-2D vs. ONT-errorCorrection and PacBio-CCS vs. PacBio-subread vs. PacBio-errorCorrected) are used. Earlier parts of the paper use abbreviations like ONT-1D or PacBio-subreads, but not here.*

**We are sorry for confusing labels in Table 2 and main text. In Table 2, “correct” means one of three SIRV annotation libraries (“correct”, “insufficient” and “over-annotated”). However, in the end of first paragraph, right column, Page 10, the word “corrected”/”correction” means the sequencing long reads that are corrected by short reads using error-correction software (e.g., LSC). We have added some annotation for Table 2 for better understanding.**

*HT (3c):  A similar statement is true for figure 5 and the corresponding text (“when using long reads only”): it is not clear if PacBio-CCS or PacBio-subreads are used (and the same for ONT) when comparing to the error-corrected subreads.*

**We are sorry for the unclear figure legend of Figure 5. The x-axis shows the strategy of isoform identification and quantification. Here, the words “correct”, “insufficient” and “over-annotated” inside the parentheses represents three different SIRV annotation libraries that were used in the "reference-annotation-guided" mode of StringTie. They do not represent the types of sequencing reads. We have modified the figure legends to clarify this issue.**

**In addition, we have updated the section “Short read and long read data processing and alignment” to describe more details about which long reads were used in the analyses. Reads used in the technical comparisons are defined specifically throughout as being either consensus or raw reads (e.g. CCS or subreads).  For the transcriptome analyses, both PacBio and ONT reads were comprised of “best reads”. These were constructed with the goal of 1) having each molecule represented in the dataset once and only once and 2) choosing the best read of each molecule for transcriptome analysis.  Below is the priority order of reads to be selected as the "best read" for each molecule in different analysis strategies:**

**PacBio (long reads only)**

1. **The best aligned CCS read (defined by the number of bases in the read mapped to the reference genome)**
2. **Otherwise, the best aligned subread**

**PacBio (long and short reads combined, Hybrid-Seq)**

1. **The best aligned CCS read with >2 passes and accuracy greater than 95 (estimated by SMRT Analysis software).  Corrected reads were not used here because the consensus is already exceeding typical short read correction.**
2. **Otherwise, the best aligned CCS read corrected by short reads.**
3. **Otherwise, the best aligned subread corrected by short reads.**

**ONT (long reads only)**

1. **The best aligned 2D read**
2. **Otherwise, the best aligned 1D template read**
3. **Otherwise, the best aligned 1D complement read**

**ONT (long and short reads combined, Hybrid-Seq)**

1. **The best aligned 2D read corrected by short reads**
2. **Otherwise, the best aligned 1D template read corrected by short reads**
3. **Otherwise, the best aligned 1D complement read corrected by short reads**

**So, for example of the “long read only” analysis of ONT, a 2D read was aligned, its best alignment would be used, and 1D reads would not be used.**

*HT (3d):  For figure 5, it is somewhat difficult to understand, what was exactly done. The authors say that, they used the “Euclidean distance” between REP and estimated REP. The way I understand it, is that the authors calculated REP and estimated REP for each transcript, and then calculated the Euclidean distance for each isoform. In this case (one dimension only) the Euclidean distance reduces to the absolute value of REP minus estimated REP. If this was done, this simpler way of saying it, is advantageous, I believe. Using the word “Euclidean distance” makes me expect multidimensionality. This would suggest that the authors have a vector of isoform expression values for each gene (or maybe multiple samples)? That would imply that the boxplots only represent 7 dots for the 7 SIRV genes…please clarify so that there is no doubt.*

**Thank you for the question.  The “Euclidean distance” is the aggregated measure of errors that are the differences between the expected relative expression percentage (REP) and observed REP.**

**We calculated “Euclidean distance” with multiple dimensions, where each transcript represents one dimension.  The expected REP of each transcript is 1/68. The observed REP was calculated by dividing a transcript  TPM (or read counts) by the sum of all observed TPMs (or read counts) of 68 SRIV transcripts. Below is the formula:**

***Total_expression = Isoform1_TPM + Isoform2_TPM + … + Isoform68_TPM***

***Euclidean_distance = sqrt((Isoform1_TPM/Total_expression-1/68)^2+(Isoform2_TPM/Total_expression-1/68)^2+…+(Isoform68_expression/Total_expression-1/68)^2) *** 

*HT (3e):  The section “Isoform Identification in hESCs by PacBio, ONT and Hybrid-Seq” is difficult to read. This may stem from the terms “full length rates” and “full-length isoform identification rates”. It is not fully clear, if they mean the same or different things; What is exactly meant? Is it “fraction of discovered annotated isoforms that are seen at least once in a full length read” or “fraction of reads that are judged as full-length” or something else? Please clarify.*

**We are sorry for the unclear description. The terms “full length rates” and “full-length isoform identification rates” mean the same things: *“fraction of discovered annotated isoforms that are seen at least once in a full length read”*. We changed “full length rates” to “full-length isoform identification rates” to for consistency. Please find the detailed definition of “full-length isoform identification rates” in Methods section (“Isoform identification in hESCs by PacBio, ONT and Hybrid-Seq”).**

*HT (3f):  Page 12, the third paragraph, regarding the discovery of isoforms with >=30 exons. The correct finding of isoforms with lots of exons of course depends on error-rate (which is linked to getting all splice sites correctly) and having long enough reads. In the absence of a size selection experiment for the Minion, one cannot prove that the observed difference between PacBio and Minion would not be rendered smaller (probably not totally removed though – because of the higher Minion error rate), with a size selection experiment for the Minion. I would mention that.*

**We agree that it is important to mention the size-selection difference in two sequencing experiments, since it could affect these numbers.  We have adjusted the manuscript text accordingly to report the observations, and not to draw conclusions about the technologies relative capabilities.**

*HT (3g):  Regarding the quantification of alternative splicing events … there are many publications that suggested exon skipping is the most frequent type of alternative splicing in humans. There are reports that have reported high occurrence of intron retention – but to my knowledge not that intron retention is more frequent than exon skipping. For example Braunschweig et al, Genome Research 2014* *^2^* *using short reads and our own paper Tilgner et al, Nature Biotech, 2015* *^1^* *using long reads. The authors could also use a minimum frequency of each single alternative event (e.g. 10% as in the papers referenced above) to distinguish splicing errors and few intermediate RNA molecules from “real” isoforms. This may change the relative abundance of each type of splicing event.*

**Thanks for your suggestions. Based on this suggestion, we calculated the minimum frequency of each single alternative splicing event and took 10% as the cut-off. The results showed that exon skipping is the most frequent AS event as the reviewer expected (see the updated Figure 6).  We have also updated our analyses in Results section “Complexity of the hESC transcriptome”.**

*HT (3h):  In the last paragraph on page 12, the word “alterative” is used. I assume this should be “alternative”. If this is a spelling mistake, there may be more.*

**Thank you for pointing out this typo.  We have fixed this in the manuscript.**

# Peer review: Referee response for version 1

**DOI:** 10.5256/f1000research.11392.r19894

**Published:** 2017-02-27

**Authors:** Jingyi Jessica Li

**Affiliations:** 1 Department of Statistics, University of California, Los Angeles, Los Angeles, CA, USA

In this paper, the authors provides comprehensive analyses to compare two third-generation sequencing technologies (PacBio and ONT) for RNA sequencing. The comparison was conducted in many aspects, including read lengths, mappability, chimeric and gapped alignments, error patterns, isoform identification, and isoform abundance estimation. To my knowledge, this paper is the first comparison of PacBio and ONT and using each of them in hybrid with Illumina, and its comparison results will provide valuable information about these two third-generation technologies to the transcriptomics field. My comments/questions about some contents in this paper are summarized below.

1. In the isoform identification task, it is unclear how the authors defined "true positive and false positive isoforms" assembled by StringTie from Illumina reads?
2. In Figure 1, why does ONT 2D have more reads than ONT 1D?
3. In the comparison of error patterns, the definition of “homopolymer pattern” is unclear.
4. In Figure 2, only the percentages of mapped reads of each read category are shown. While this is important information, it would be also important to know the absolute numbers of mapped reads in each category.
5. In Figure 3, the row containing labels “A C G T ” above the insertion row should be better placed above the mismatch row.
6. In Table 2, the top row labeling is confusing. It would be clearer to remove "Over-annotated library (100)", "Correct library (68)", and "Insuf cient library (43)" from the top row. Also why does the "Illumina+Insufficient" row have one additional cell?
7. In Figure 4, it would be better to make the circles in Venn diagrams proportional to the numbers?
8. It is unclear why the authors included the insufficient annotation and the overannotated cases in the study of isoform identification and isoform abundance estimation. Since they are only applicable to the Illumina data using StringTie but not relevant to the PacBio or the ONT data, including them seems deviation from the theme of the paper.
9. In Figure 7d, are the seven novel isoforms verified?

I have read this submission. I believe that I have an appropriate level of expertise to confirm that it is of an acceptable scientific standard, however I have significant reservations, as outlined above.

# Response

**Published:** 2017-06-07

**Authors:** Kin Fai Au

**Affiliations:** University of Iowa, USA

**We greatly appreciate your time and thoughtful questions and critiques of our manuscript “Comprehensive comparison of PacBio and Oxford Nanopore Technologies and their applications to transcriptome analysis.”  These are addressed in this point by point response and in the corresponding manuscript revisions.**

*JJL: In this paper, the authors provides comprehensive analyses to compare two third-generation sequencing technologies (PacBio and ONT) for RNA sequencing. The comparison was conducted in many aspects, including dread lengths, mappability, chimeric and gapped alignments, error patterns, isoform identification, and isoform abundance estimation. To my knowledge, this paper is the first comparison of PacBio and ONT and using each of them in hybrid with Illumina, and its comparison results will provide valuable information about these two third-generation technologies to the transcriptomics field. My comments/questions about some contents in this paper are summarized below.*

*JJL (1): In the isoform identification task, it is unclear how the authors defined "true positive and false positive isoforms" assembled by StringTie from Illumina reads?*

**Thank you for your question.  Our original manuscript did not adequately describe the criteria used in this analysis and has been modified accordingly in the revision (see the second paragraph in Methods “Isoform identification in SIRVs by Illumina, PacBio, ONT”):  “For all SIRV isoforms, we classified them into two groups: 1) true positive if the isoform was annotated by SIRV “correct” annotation library; and 2) false positive if not.**

*JJL (2): In Figure 1, why does ONT 2D have more reads than ONT 1D?*

**We apologize if the axis labeling and scaling of Figure 1 made this point unclear; raw counts associated with this figure are available in the Supplemental Table 2.  As expected, ONT 2D have less reads (289430) than the ONT 1D reads (339651). The Figure 1 legend has been modified to appropriately refer readers to Supplementary Table 2.**

*JJL (3): In the comparison of error patterns, the definition of “homopolymer pattern” is unclear*

**Thank you for give us a chance to clarify definitions of “loose” and “tight” homopolymer patterns.  If an error rate is much higher when both surrounding bases are the same as the mismatched, inserted or deleted bases, then it indicates that these errors are mostly occurring in a homopolymer runs.  In the “loose” homopolymer error pattern, the error rate is high if either surrounding bases are the same as the mismatched, inserted or deleted bases. The context requirement is “looser” than “tight” homopolymer patterns. This is observed as the cross-shaped higher error rates (in the context of Figure 3).**

*JJL (4): In Figure 2, only the percentages of mapped reads of each read category are shown. While this is important information, it would be also important to know the absolute numbers of mapped reads in each category.*

**We agree that total number of aligned reads represented in each category would be a very useful addition, and have updated Figure 2 accordingly and added to the figure legend.**

*JJL (5): In Figure 3, the row containing labels “A C G T ” above the insertion row should be better placed above the mismatch row.*

.

**Thank you for the suggestion.  To improve the visual cues in the figure, we have filled out the labeling in Figure 3 around the mismatch patterns.**

*JJL (6): In Table 2, the top row labeling is confusing. It would be clearer to remove "Over-annotated library (100)", "Correct library (68)", and "Insuf cient library (43)" from the top row. Also why does the "Illumina+Insufficient" row have one additional cell?*

**Thanks for your suggestion. We revised Table 2 to clearly show our results on isoform identification.**

*JJL (7): In Figure 4, it would be better to make the circles in Venn diagrams proportional to the numbers?*

**Yes, we agree with you. For Figure 4e and 4f, the circles in Venn diagrams were made to be proportional to the numbers.**

*JJL (8): It is unclear why the authors included the insufficient annotation and the overannotated cases in the study of isoform identification and isoform abundance estimation. Since they are only applicable to the Illumina data using StringTie but not relevant to the PacBio or the ONT data, including them seems deviation from the theme of the paper.*

**For isoform identification, a “reference-annotation-guided” mode is recommended by most  short read-based method (e.g. Cufflinks and StringTie). The performance could strongly rely on the reliability and completeness of the reference annotation library. To consider different scenarios, we included three types of reference annotation libraries in the comparison. In detail, we want to prove two points:**

**1) First, for most non-model organisms, isoform annotation libraries are incompletely annotated and thus insufficient for transcriptome analysis. Recovering un-annotated isoforms that are expressed in the given sample is therefore challenging. As shown in Table 2, StringTie (by Illumina data and “insufficient library”) only rescued 5 of 25 un-annotated but truly-expressed isoforms. Second, for well-studied species like human, not all annotated isoforms are expressed in a given sample. Thus, the isoform annotation libraries are usually “over-annotated” for a given sample (e.g., a specific cell line or tissue). Using the “over-annotated library” with 32 unexpressed isoforms, StringTie incorrectly assembled 15 unexpressed isoforms, which highly increased the false positive rate.  Therefore, short read-based strategies have an inherent disadvantage to long read-based strategies, and prediction alone is insufficient to overcome this.**

**2) Long read sequencing technologies (PacBio or ONT) can directly detect expressed isoforms. As shown in Table 2, PacBio and ONT detected 67 and 68 expressed isoforms, respectively.  Therefore, both long read-based strategies overcome shortcomings of prediction through direct detection.**

*JJL (9): In Figure 7d, are the seven novel isoforms verified?*

**In this study, we did not verify identified novel isoforms. In our future work, we will verify these novel isoforms, especially for those isoforms that are associated with embryonic stem cell identity. To increase the reliability of novel isoforms, we have updated Figure 7d and only retained those novel isoforms that are supported by both PacBio and ONT full-length long reads. The number of supporting long reads can be found in updated Supplementary Table 3.  The manuscript has also been updated to reflect this change (see Results “Functional analysis of identified isoforms in hESCs”).**
